# Mechanochemistry: Fundamental Principles and Applications

**DOI:** 10.1002/advs.202403949

**Published:** 2024-08-29

**Authors:** Liang Dong, Luofei Li, Huiyan Chen, Yi Cao, Hai Lei

**Affiliations:** ^1^ Collaborative Innovation Center of Advanced Microstructures National Laboratory of Solid State Microstructure Department of Physics Nanjing University Nanjing Jiangsu 210093 P. R. China; ^2^ School of Physics Zhejiang University Hangzhou Zhejiang 310027 P. R. China; ^3^ Institute of Advanced Physics Zhejiang University Hangzhou Zhejiang 310027 P. R. China

**Keywords:** biomechanochemistry, force, mechanism, mechanochemistry, single molecule, soft materials

## Abstract

Mechanochemistry is an emerging research field at the interface of physics, mechanics, materials science, and chemistry. Complementary to traditional activation methods in chemistry, such as heat, electricity, and light, mechanochemistry focuses on the activation of chemical reactions by directly or indirectly applying mechanical forces. It has evolved as a powerful tool for controlling chemical reactions in solid state systems, sensing and responding to stresses in polymer materials, regulating interfacial adhesions, and stimulating biological processes. By combining theoretical approaches, simulations and experimental techniques, researchers have gained intricate insights into the mechanisms underlying mechanochemistry. In this review, the physical chemistry principles underpinning mechanochemistry are elucidated and a comprehensive overview of recent significant achievements in the discovery of mechanically responsive chemical processes is provided, with a particular emphasis on their applications in materials science. Additionally, The perspectives and insights into potential future directions for this exciting research field are offered.

## Graphical abstract

In this review, the physicochemical principles of mechanochemistry are first clarified. Based on this foundation, the research basis and cutting‐edge scientific achievements in four areas are introduced: solid‐state organic chemistry, polymers, interface science, and biomechanochemistry. Special emphasis is placed on the applications of mechanochemistry in materials science. Furthermore, the perspectives and insights on the research challenges and potential future directions are offered.

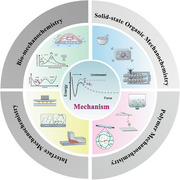

## 1 Introduction

Mechanochemistry refers to the coupling of chemical reactions with mechanical forces.^[^ ^1^ ^]^ Given that mechanical stresses are ubiquitously encountered by various materials, mechanochemistry finds broad applications across materials science. For instance, methods such as ball milling^[^ ^2^, ^3^, ^4^ ^]^ and twin‐screw extrusion^[^ ^5^, ^6^, ^7^, ^8^ ^]^ have been utilized to synthesize metallic nanoparticles, catalysts and polymer powders in a solvent‐free and eco‐friendly manner. Synthetic mechano‐responsive building blocks, or mechanophores, have been employed to sense mechanical forces and detect defects in elastomers.^[^ ^9^ ^]^ Additionally, dynamic covalent bonds have been incorporated into hydrogel networks to effectively dissipate mechanical energy and enhance toughness.^[^ ^10^, ^11^, ^12^, ^13^, ^14^, ^15^, ^16^ ^]^ Ultrasonication in mechanochemistry enhances material synthesis,^[^ ^17^, ^18^, ^19^ ^]^ catalysis and degradation^[^ ^20^, ^21^, ^22^ ^]^ for efficient material creation, nanoparticle dispersion.^[^ ^23^, ^24^, ^25^ ^]^ and crystal engineering.^[^ ^26^, ^27^, ^28^, ^29^, ^30^ ^]^ In addition, ultrasound has been applied to cut and functionalize carbon nanotubes in solution.^[^ ^26^, ^27^, ^28^, ^29^, ^30^ ^]^ In the realm of biomaterials, mechanical properties play a crucial role and can regulate cell spread, proliferation, migration, and stem cell differentiation.^[^ ^31^, ^32^, ^33^, ^34^, ^35^, ^36^, ^37^, ^38^, ^39^, ^40^, ^41^, ^42^, ^43^ ^]^ Exploiting mechanochemistry in materials science not only enhances the mechanical properties of materials but also leads to the development of materials with novel mechanical responses.^[^ ^44^, ^45^, ^46^, ^47^, ^48^, ^49^, ^50^, ^51^ ^]^ These investigations, in turn, facilitate the fundamental understanding of mechanochemical processes and inspire the design of mechanophores.^[^ ^52^ ^]^

Mechanochemistry has emerged as an interdisciplinary field, drawing contributions from various groups of scientists.^[^ ^53^, ^54^, ^55^, ^56^, ^57^ ^]^ Despite that advances in mechanochemistry have been extensively reviewed, these reviews mainly emphasize on phenomenology. Empirical rules derived from specific aspects of mechanochemistry may not always be applicable across different contexts, making it challenging to link macroscopic mechanical effects to force‐dependent structural changes at the molecular level and predict outcomes accurately. Recently, Akbulatov and Boulatov provided excellent reviews on the interpretational frameworks of polymer mechanochemistry,^[^ ^58^, ^59^, ^60^ ^]^ laying a solid foundation for understanding the mechanical aspects of this field. Building upon their work, we aim to further expand our understanding of mechanical forces in mechanochemistry, encompassing a broader range of directions and applications. By delving into the molecular mechanisms underlying mechanochemical processes, we strive to advance the field and facilitate the quantitative prediction of outcomes under mechanical forces.

### 1.1 Physical Principles of Mechanochemistry

**Figure 1:**
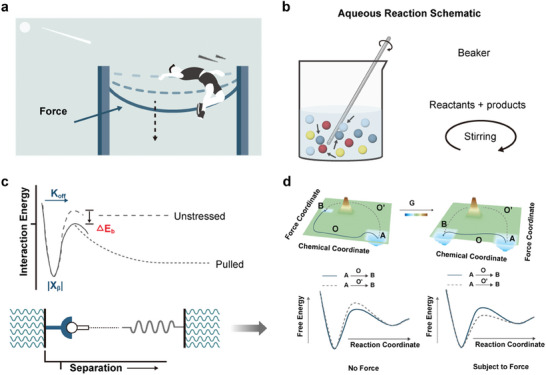
Physical principles of mechanochemistry. a) The activation energy can be reduced by applying force so that the reaction can occur, just as a crossbar can be bent by force. b) Mechanochemistry is the reaction of particles in directed collisions through the application of a force. c) 1‐D free‐energy mapping can provide an explanation for mechanochemical reactions. d) In 2‐D free‐energy mapping, mechanochemistry can induce a chemical reaction to proceed via a different pathway.

The kernel of chemical reactions is the formation and rupture of chemical bonds. In 1889, Arrhenius proposed a relationship between temperature and chemical reaction rates, derived from numerous experiments.^[^ ^61^ ^]^ This relationship is encapsulated in an empirical formula *k*  =  *Ae* ^−*Ea*/*RT*^, where *k* represents the reaction rate constant, *R* denotes the molar gas constant, *T* stands for the temperature in Kelvin, *E_a_* signifies the apparent activation energy and *A* represents the pre‐exponential factor. The activation energy is the minimum energy threshold that must be surmounted to initiate a chemical reaction. Chemical reactions are like high jumps. If we compare the activation energy to the height of the bar, when the activation energy is mechanically lowered, it becomes easier to overcome the energy barrier, making the reaction more likely to occur (**Figure** **1**a). Due to the conservation of energy, more kinetic energy can surmount higher obstacles. Similar principles also apply in chemistry. If the free energy of reactants exceeds the activation energy of a reaction, they can surmount the kinetic barrier and transition into products. The pre‐exponential factor, also known as the frequency factor, quantifies the frequency of collisions between reactant molecules and the likelihood of achieving the correct molecular orientation conducive to the reaction. Often regarded as an entropic factor, it emphasizes the stochastic nature of collisions and their potential to yield a reaction. Clearly, both the pre‐exponential factor and the activation energy play pivotal roles in regulating chemical reactions.

Depending on the specific methods employed, mechanical energy can induce two primary effects: altering the motion of molecules or creating intramolecular strains. Consequently, the influence of force on reactions manifests in two distinct ways. First, it promotes the effective collision probability. Mechanical force ensures thorough mixing of reactants, increasing collision frequency and promoting uniform reaction conditions. This intense mixing prevents concentration gradients and dissipates localized heat, enhancing overall reaction rates (Figure 1b). Also, it breaks down larger particles into smaller ones, increasing surface area and creating more opportunities for reactant molecules to collide. Furthermore, mechanical stress generates structural defects and reactive sites, lowering activation energy and making collisions more effective. This phenomenon is well understood within a simplified 1‐D free energy landscape, initially described by Eyring and further elaborated by Bell^[^ ^62^ ^]^ and Evans^[^ ^63^ ^]^ (Figure 1c). This model predicts the linear dependence of the survival probability density on the logarithm of the stretching force loading rate, known as the Bell–Evans model. The interaction rate α(*F*) can be described as:

(1) $$\alpha \left(F\right)\;=\alpha_{0}\;e^{\frac{F\Delta x}{k_{B}T}}$$

Here, *k_B_* represents the Boltzmann constant, α_0_ denotes the characteristic spontaneous rate of the reaction in the absence of force, and Δ*x* signifies a characteristic spatial scale of the interaction landscape. This model was refined by Boulatov to include the second‐order Taylor expansion term of the activation energy and by Dudko, Hummer and Szabo to account for the curvature of the free energy landscape.^[^ ^64^ ^]^ Notably, when considering the effect of force on the free energy within a 2‐D landscape, it becomes apparent that force can alter the reaction pathway and potentially yield entirely different products^[^ ^17^, ^65^, ^66^ ^]^ (Figure 1d). According to the first law of thermodynamics, mechanical work can also generate heat. Therefore, when examining the impact of mechanical forces on a chemical reaction, it is imperative to carefully distinguish it from the heat effect.

In the subsequent sections, we delineate the latest advancements in the realm of mechanochemistry within the framework of the Arrhenius equation. We elucidate how the influence of forces manifests differently across various facets of mechanochemistry, encompassing solid‐state organic mechanochemistry, polymer mechanochemistry, surface mechanochemistry, and mechanobiochemistry. Furthermore, we underscore the primary challenges encountered in this burgeoning and exciting field. In conclusion, we outline potential future directions in mechanochemistry, offering insights into how ongoing research efforts can propel this field forward.

## 2 Solid‐State Organic Mechanochemistry

One of the most compelling and intuitive realms where mechanochemistry finds application lies within solid‐state organic chemistry. Although the rudimentary act of grinding herbs in a mortar and pestle in ancient times can be regarded as an early instance of mechanochemical action, its significant advancement has predominantly occurred in recent years with the advent of novel techniques and instrumentation. In addition to traditional grinding, contemporary methodologies such as uniaxial compression across an interface have emerged, offering innovative means to exert forces on molecules, thereby expanding the horizons of organic chemistry.^[^ ^67^, ^68^ ^]^ These mechanochemical processes are facilitated through various apparatuses, including batch preparation equipment such as planetary mills, vibrating screens, and grinders, as well as continuous preparation machinery such as twin‐screw extruders. Through the utilization of these methodologies, there is a notable reduction, and in some cases, complete elimination of solvent usage in solid‐state organic mechanochemistry. Therefore, this approach has garnered recognition as an excellent method of Green Chemistry. Furthermore, the significance of solid‐state organic mechanochemistry has been underscored by its inclusion in the International Union of Pure and Applied Chemistry (IUPAC)’s list of 10 world‐changing technologies.

### 2.1 Solid‐State Organic Mechanochemistry Methods

**Figure 2:**
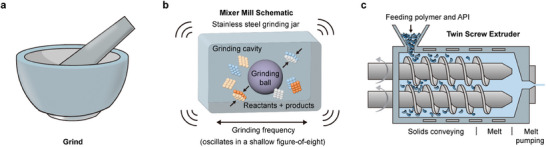
Scheme of different technologies in solid‐state organic mechanochemistry. a) Mortar provides shear and pressure. b) Ball mills not only pulverize samples but also provide more powerful compression. c) Twin‐screw extrusion enables the preparation of compression molded fiber materials.

The pulverization of solid samples or the genesis of emergent substances through compression grinding has been a practice since antiquity^[^ ^69^ ^]^ (**Figure** **2**a). This ancient practice laid the foundation for primitive mechanochemistry, rooted in the reconstruction of intermolecular interactions. Among the tools favored by mechanochemical researchers, the ball mill stands out prominently. These devices, available in various sizes and designs, feature one or more loose balls that pulverize materials within them as the vessel is either shaken or rotated (Figure 2b). Consequently, both compressive and shear forces are exerted on the molecules contained within. Commercially utilized for diverse purposes beyond synthesis for well over a century, ball mills have found widespread application in numerous industries, including mining, cement, chemical, pharmaceutical, wood, and ceramics industries.^[^ ^70^, ^71^ ^]^ Additionally, they are extensively employed for mixing, blending, and mechanical alloying.^[^ ^72^, ^73^, ^74^ ^]^ However, it is only in recent years that the application of ball mills for chemical synthesis has gradually gained traction.^[^ ^75^, ^76^, ^77^, ^78^ ^]^

Indeed, the ball mill is not the only means to induce molecular reactions with mechanical forces; the twin‐screw extruder is another suitable option. This apparatus comprises two elongated screws positioned closely within a barrel, never making direct contact (Figure 2c). Samples are introduced at the top of the screws and traverse downward as the screws rotate, encountering designed screw profiles that induce vigorous mixing at specific points along their length. Similar to ball mills, extruders apply both compressive and shear forces to the molecules within them. One key advantage of twin‐screw extruders lies in their capability for continuous product manufacturing.^[^ ^79^, ^80^, ^81^, ^82^, ^83^ ^]^ In contrast, ball mills are primarily suited for batch production.

### 2.2 Mechanism of Solid‐State Organic Mechanochemistry

The underlying mechanism of solid‐state organic mechanochemistry differs from the chemical reactions in solutions. In solutions, the collision of reactant molecules is influenced by diffusion, molecular charge properties, and affinity. For instance, molecules with opposing charges or varying affinities may experience reduced collision rates due to repulsion or steric hindrance, which can impede reaction progress. However, solid‐state organic mechanochemistry circumvents these limitations probably by formation of liquid or melt phase, which imbues the individual molecules with the required mobility for productive (or reactive) collision, intervenes, allowing rapid reaction between the two solid reagents.^[^ ^84^ ^]^ In this context, the application of external force brings reactant molecules into closely proximity, overcoming diffusion barriers and promoting intermolecular interactions. As described by the Arrhenius equation, the mechanical force in solid‐state organic mechanochemistry accelerates the reaction rate k, and facilitates efficient reactions, regardless of molecular charge properties or affinity differences. Furthermore, bypassing diffusion‐related constraints and promoting direct molecular interactions, mechanochemical approaches provide new pathways for synthesizing organic molecules and materials with improved reactivity, selectivity, and sustainability. This innovative approach expands the horizons of organic synthesis and lays the foundation for the advancement of sustainable and environmentally friendly chemical processes. However, there remains a significant gap in understanding the detail mechanisms in solid‐state organic mechanochemistry. Without a comprehensive grasp of how mechanical energy translates into chemical reactivity, predicting the outcomes of reactions under these conditions is challenging.

### 2.3 Solid‐State Organic Mechanochemistry Applications

**Figure 3:**
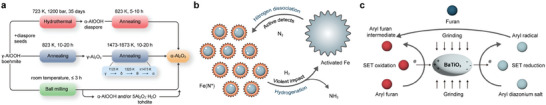
Applications of solid‐state organic mechanochemistry. a) Alteration of the crystal lattice structure using ball mills. b) Utilizing stress as a catalyst to improve the reaction efficiency. Reproduced with permission.^[^ ^91^ ^]^ Copyright 2020, Springer Nature Limited. c) Reactions are carried out by inputting activation energy into the reactants with the help of extrusion. Reproduced with permission.^[^ ^92^ ^]^ Copyright 2019, AAAS.

With the advancement of solid‐state organic mechanochemistry, various appealing products, including amino acids, hydrazine, nitrones, peptides, and classical organic reactions such as Suzuki coupling and click chemistry, have been successfully synthesized utilizing ball milling machines.^[^ ^85^ ^]^ For instance, a straightforward mechanochemical grinding method employing ball mills has yielded high‐surface‐area corundum (α‐Al_2_O_3_) through the dehydration of boehmite (γ‐AlOOH) at room temperature^[^ ^86^, ^87^ ^]^ (**Figure** **3**a). The impact of milling on surface energy shifts the stability from boehmite to α‐Al_2_O_3_ and leads to the synthesis of nanometer‐sized α‐Al_2_O_3_ nanoparticles with an average diameter of ≈13 nm and surface areas reaching ≈140 m^2^ g^−1^. The exceptional mechanical stability and high surface area of corundum render it suitable for various applications, such as catalysts, catalyst carriers, and sorbents. Additionally, it is feasible to convert boehmite into other crystal structures through ball milling.^[^ ^88^, ^89^ ^]^

Achieving scalable and cost‐effective production of graphene nanosheets (GnPs) stands as a pivotal challenge for their commercial viability. A notable breakthrough has been made in the preparation of various edge‐functionalized GnPs (EFGnPs) via a straightforward mechanochemical reaction.^[^ ^90^ ^]^ Ball milling of graphite alongside a target substance (Substance X, which represents nonmetals, halogens, semimetals, or metalloids) enables the production of EFGnPs in an environmentally friendly manner. This method harnesses kinetic energy to dismantle the graphitic C─C bonds under dry, solvent‐free conditions, yielding activated carbon. The activated carbon exhibited enhanced affinity for Substance X, facilitating the formation of graphitic C─X bonds along fracture edges and the layering of graphite sheets into EFGnPs. Unlike the preparation of graphene oxide (GO) and reduced graphene oxide (rGO), the synthesis of EFGnPs eliminates the need for toxic chemicals such as corrosive acids and hazardous reducing agents. Furthermore, the resulting EFGnPs maintain high crystallinity in the substrate region owing to their edge‐selective functionalization. With an array of edge X groups at disposal for selective utilization, the potential applications of EFGnPs are boundless, particularly as metal‐free carbon‐based electrocatalysts for dye‐sensitized solar cells (DSSCs) in cobalt and iodine electrolytes.

Ball mills have also been extended to the synthesis of ammonia (NH_3_), a crucial feedstock for fertilizers, pharmaceuticals, and fine chemical engineering. Conventionally, ammonia synthesis involves the Haber–Bosch process, which requires high temperatures (400–500 °C) and pressures (above 100 bar) with N_2_ and H_2_ as the starting materials. However, based on mechanochemistry with ball milling, Jong‐Beom Baek et al. proposed a novel approach for synthesizing ammonia from nitrogen under mild conditions (45 °C, 1 bar) using iron powder as a catalyst^[^ ^77^ ^]^ (Figure 3b). Nitrogen molecules are adsorbed onto these defects ([Fe(N^*^)]), facilitating the dissociation of nitrogen molecules. Subsequently, nitrogen atoms undergo hydrogenation to form NH_x_^*^ (x = 1–3), with NH_x_^*^ desorbing from the catalyst surface to yield the final product, ammonia. The energy transfer generated by dynamic relaxation during collisions aids in the desorption of these intermediates. Based on this principle, the final concentration of obtained ammonia can reach up to 82.5 vol%, significantly surpassing that achievable by the conventional Haber method and electrochemical approaches. This work serves as a direct demonstration of the advantageous capabilities of mechanochemistry.

Inspired by the distinctive characteristics of photoredox systems that rely on light irradiation and the efficacy of ball milling in mechanochemistry, a novel approach has emerged: the redox activation of small organic molecules in response to mechanical energy applied through the piezoelectric effect. This complementary method represents a significant advancement in the field. A notable experiment demonstrated the mechanoredox activation of various aryl diazonium salts through applied mechanical force, facilitating arylation and borylation reactions. By mechanically agitating a mixture comprising aryl diazonium salt, furan, and BaTiO_3_, the BaTiO_3_ particles undergo deformation, transitioning into a charge‐separated state capable of acting as both an oxidant and a reductant simultaneously. Subsequently, this leads to the arylation and borylation of the aryl diazonium salts^[^ ^77^, ^92^ ^]^ (Figure 3c). This finding underscores the pivotal role of these components in facilitating the mechanoredox process. This groundbreaking approach establishes a new mechanochemical route for robust and sustainable synthesis, with broad applicability across a wide range of organic redox reactions.

In contrast to ball milling, there is growing interest among scientists in twin‐screw extruders, which belongs to a class of continuous processing technique wherein materials are compressed through confined spaces. Notably, the biopharmaceutical company Amgen has reported advancements in cocrystal synthesis by harnessing the potential of twin‐screw extruders, enabling the large‐scale production of medicines for pain therapy.^[^ ^93^, ^94^ ^]^ This represents a significant milestone, as it marks the first instance of synthesizing hundred‐gram scale products utilizing solid‐state mechanochemistry. While multikilogram‐scale mechanochemical synthesis of drug carrier composites utilizing ball milling has been reported, twin‐screw extruders have also demonstrated remarkable effectiveness in synthesizing various covalently bonded metal–organic frameworks (MOFs) and metal complexes. These processes often require minimal or no added solvent and yield products of high purity, yield, and crystallinity.^[^ ^95^, ^96^, ^97^, ^98^, ^99^, ^100^ ^]^ The findings from these studies herald a new era in green, sustainable, and solvent‐free chemical techniques, offering promising avenues for advancing environmentally friendly synthesis methods.

Indeed, while significant strides have been made in solid‐state mechanochemistry, there remains a pressing need for further research to deepen our understanding of its underlying mechanisms. This lack of comprehensive knowledge poses challenges in effectively designing chemical processes and accurately predicting product outcomes. To address this gap, continued investigation into the fundamental principles governing mechanochemical reactions is essential. By elucidating the intricate mechanisms at play, researchers can enhance the efficacy of chemical processes and refine predictive models, ultimately advancing the field of mechanochemistry toward more precise and controlled synthesis methodologies.

## 3 Polymer Mechanochemistry

**Figure 4:**
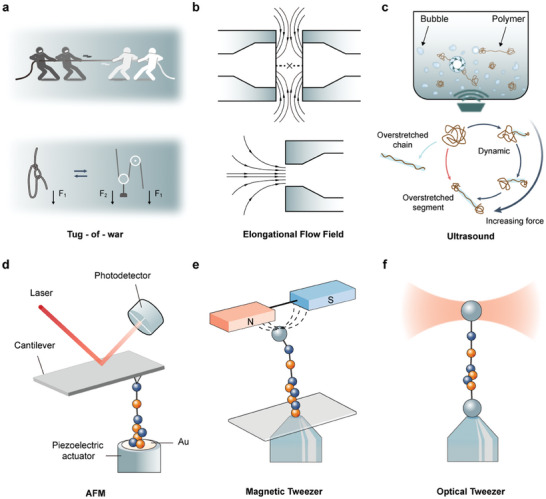
Techniques for polymer mechanochemistry study. a) Tug‐of‐war and sliding knot to describe the polymer mechanochemistry. b) Schematic of the representative elongational flow fields. c) Illustration of polymer stretching through ultrasonication and how polymers respond to cavitation. Reproduction with permission.^[^ ^102^ ^]^ Copyright 2023, Springer Nature Limited. d) AFM‐based single‐molecule force spectroscopy. e) MT‐based single‐molecule force spectroscopy. f) OT‐based single‐molecule force spectroscopy.

Polymers pervade various aspects of daily life, from consumer products to medical devices, owing to their exceptional mechanical properties, such as high strength, toughness, elasticity, and processability. Consequently, the field of polymer science has become a focal point for mechanochemistry, giving rise to polymer mechanochemistry. Unlike solid‐state organic mechanochemistry, which primarily centers on chemical reactions occurring within solid materials, polymer mechanochemistry delves into understanding and harnessing physicochemical reactions within polymer chains induced by stretching caused by interactions between the chains and their environment.^[^ ^60^, ^101^ ^]^ Thus, polymer mechanochemistry can be likened to a game of tug‐of‐war, in which the mechanophore is more like a knot made of rope, as illustrated in **Figure** **4**a. The key to comprehending the physical mechanisms in polymer mechanochemistry is deciphering the transduction of forces within polymer chains and their regulation of mechanosensitive groups known as mechanophores. These mechanophores play a crucial role in sensing and responding to mechanical stimuli, thereby influencing the behavior and properties of polymer materials.

### 3.1 Applying Mechanical Force Methods

#### 3.1.1 Early Mechanical Force Methods used in Polymer Mechanochemistry

In the earlier stages of its development, polymer mechanochemical reactions could be initiated through elongational flow fields and ultrasound. Elongational flow fields, the easiest way to think of applying forces to polymer chains^[^ ^103^, ^104^, ^105^ ^]^ (Figure 4b), involve a velocity gradient along the direction of flow, stretching polymers far from equilibrium and potentially leading to bond scission. However, this method typically requires high‐molecular‐weight polymers for investigation and lacks controllability, thus limiting the study of mechanophores. Ultrasound, on the other hand, can be utilized to accelerate and modify chemical reactions in polymer solutions.^[^ ^23^, ^106^, ^107^, ^108^, ^109^ ^]^ Recent studies show that polymers respond to cavitation in a way that is different from conventional thinking: the unfolding process of polymers progresses over time in a wave‐like state rather than fully unfolding^[^ ^102^, ^110^ ^]^ (Figure 4c). However, the accurate force calibration of ultrasonication is challenge and it is difficult to quantitative manipulate individual polymer. Additionally, ultrasonication can generate unwanted side reactions or alter the desired chemical pathways.^[^ ^111^, ^112^, ^113^ ^]^

#### 3.1.2 Single‐Molecule Force Spectroscopy (SMFS) Techniques

As polymer mechanochemistry has evolved into a precision discipline for inducing molecular reconfiguration in response to macroscopic mechanical influences, single‐molecule force spectroscopy (SMFS) techniques have emerged (Figure 4d–f).^[^ ^114^ ^]^ The most commonly used force spectroscopy techniques are atomic force microscopy^[^ ^115^, ^116^, ^117^, ^118^, ^119^, ^120^, ^121^, ^122^, ^123^ ^]^ (AFM, Figure 4d), magnetic tweezers^[^ ^124^, ^125^, ^126^, ^127^, ^128^, ^129^, ^130^, ^131^ ^]^ (MT, Figure 4e), acoustic tweezers^[^ ^132^, ^133^, ^134^, ^135^, ^136^, ^137^ ^]^ (AT), Biomembrane force probe^[^ ^138^, ^139^, ^140^, ^141^, ^142^, ^143^, ^144^, ^145^, ^146^, ^147^ ^]^ (BFP) and optical tweezers^[^ ^148^, ^149^, ^150^, ^151^, ^152^, ^153^, ^154^ ^]^ (OT, Figure 4f), which enable precise manipulation and measurement of forces and lengths at the single‐molecule level. AFM can directly apply force to polymers through a linked cantilever tip, while OT, AT, BFP, and MT manipulate polymers by applying optical trap force, sound pressure, force transmitters (usually cell‐sized, such as vesicles, liposomes, or red blood cells) and magnetic force to linked microspheres, respectively. Due to their high accuracy in force measurement (picometer) and length measurement (nanometer), SMFS techniques are widely employed to investigate the mechanisms of polymer mechanochemistry at the single‐molecule level.

### 3.2 Mechanophores

Mechanophores represent a class of small molecules characterized by their ability to elicit a physical or chemical response upon exposure to mechanical forces, typically leading to structural modifications such as conformational alterations or bond cleavage. Over the past two decades, the synthesis of more than 100 mechanophores has been achieved, showcasing a remarkable diversity in both structure and function. These mechanophores are widely used in the realm of polymer science, where they are strategically incorporated into polymer chains to enable responsive behavior upon the application of mechanical forces, thereby facilitating the design of advanced polymer materials.^[^ ^155^, ^156^, ^157^, ^158^, ^159^, ^160^ ^]^ Consequently, it is imperative to delve into the underlying mechanisms governing mechanophore response to elucidate their functionality with precision.

#### 3.2.1 Force Response Mechanisms of Mechanophore

**Figure 5:**
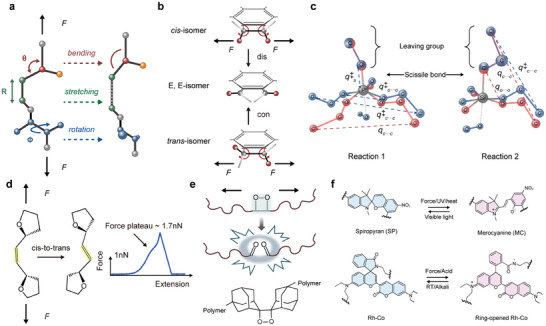
Mechanophores and their structural transformation by tension. a) Examples of the deformation of a portion of a polypropylene chain: valence angle bending (Δθ, blue), bond stretching (ΔR, green), and torsional angle rotation (Δφ, red). b) Activation by mechanical forces is predicted to induce formal disrotatory ring opening in the cis isomer and formal conrotatory ring opening in the trans isomer, such that both isomers are predicted to yield the same E, E‐oQDM intermediate. These predictions have been tested experimentally. c) Because the pulling direction is not parallel to the C═C double bonds in the polymer, stretching the polymer not only provides tension to lower the transition barrier but also provides torsion to facilitate the rotation of cis C═C bonds. d) bis(Adamantyl)−1,2‐dioxetane stress causes a flip in the spatial structure, which promotes luminescence. Reproduced with permission.^[^ ^171^ ^]^ Copyright 2016, ACS Publication. e) The four‐membered dioxyl ring of bis‐adamantyl‐1,2‐dioxetane breaks when stretched, leading to the generation of faint blue light, and the energy generated can excite other molecules to fluoresce. Reproduced with permission.^[^ ^172^ ^]^ Copyright 2012, Springer Nature Limited. f) Fluorescent molecules can undergo reversible ring‐opening reactions with physical (spiropyran, top) or chemical (lactone, bottom) stimulation. Reproduced with permission.^[^ ^173^, ^174^ ^]^ Copyright 2023, Wiley.

The mechanical responses exhibited by mechanophores can be broadly categorized into three distinct types: angle bending, bond stretching leading to scission, and bond rotation, as depicted in **Figure** **5**a. Typically, bending and stretching phenomena are interrelated when mechanical force is applied to mechanophores. A notable example illustrating this interplay is observed in 1,2‐disubstituted benzocyclobutenes (BCB) through ultrasonication^[^ ^161^, ^162^ ^]^ (Figure 5b). Here, mechanical forces act upon the trans BCB unit, inducing deformation in bond lengths and angles, consequently lowering the barrier for a formal conrotatory process and expediting the subsequent ring cleavage reaction. Conversely, for the cis isomer, mechanical force influences the BCB unit to reduce the barrier for a formal disrotatory process, distinct from the thermally allowed conrotatory ring opening. This highlights how applied mechanical force can selectively bias the reaction pathway by directly modulating the molecular potential energy surface.

The majority of mechanophores are typically characterized by the presence of mechanically labile bonds or strained rings, which endow them with unique mechanical responses beyond simple bond scission.^[^ ^163^, ^164^, ^165^, ^166^ ^]^ Through the integration of SMFS and quantitative calculations, previous studies have revealed that mechanical force can induce the rotation of C═C bonds, leading to *cis*‐to‐*trans* isomerization in polymers possessing specific structural motifs^[^ ^167^, ^168^, ^169^ ^]^ (Figure 5c). C═C bonds are ubiquitous in both natural and synthetic polymers and are conventionally regarded as mechanically inert due to their high bond energy, thus not contributing significantly to polymer elongation. Density functional theory (DFT) calculations have predicted that stretching *cis*‐1,4‐polyisoprene or polybutadiene would only lead to the breaking of C═C bonds at tensile forces of 6.8 and 7.2 nN, respectively.^[^ ^170^ ^]^ However, no mechanical *cis*‐to‐*trans* isomerization was observed before the rupture of the single C═C bond. In contrast, our experiments have directly observed *cis*‐to‐*trans* isomerization occurs prior to backbone breakage of the polymer chain, manifesting at a remarkably short timescale of 1 ms and a transition force of ≈1.7 nN.^[^ ^163^ ^]^ Notably, the force‐free transition state exhibits a C─C═C─C dihedral angle of ≈76°, deviating from the typical 0° or 180° for C═C bonds in the cis or trans conformation, respectively, suggesting the rotation of C═C bonds under stretching conditions. Given that the cis C═C bonds within the polymer are not perfectly aligned with the direction of the applied force, both tension and rotation are generated during stretching. Tension serves to reduce the energy barrier, resulting in a significantly lower engery requirement for cis‐to‐trans rotation in our polymer system. This observation challenges the conventional notion that C═C bonds are relatively stable under thermodynamic conditions, highlighting the potent influence of mechanical force in activating reactions otherwise considered “intractable” in thermally activated processes. Furthermore, this demonstration holds promise for substantially expanding the repertoire of available mechanophores.

Furthermore, an intriguing experiment revealed mechanochemical behaviors distinct from those of force‐accelerated bond scission^[^ ^171^, ^175^ ^]^ (Figure 5d). Under mechanical stretching, phosphotriesters experience accelerated dissociation of the unloaded phosphorus‐oxygen bond orthogonal to the pulling axis, whereas stretching of organosiloxanes impedes dissociation of the aligned loaded silicon‐oxygen bonds. This discrepancy in behavior originates from the structural preferences governing nucleophilic displacements and the geometry of the transition state. In phosphates, the applied force acts across the termini of the spectator alkoxy moieties, leading to an increase in the O–P–O angle, and the molecule elongates progressively along the reaction path (dashed arrows in Figure 5d). Consequently, siloxanes initially contract along the pulling axis, leading to destabilization when subjected to tensile force before elongating and ultimately breaking. This intricate interplay has facilitated the development of novel mechanophores such as pterodactylane,^[^ ^176^ ^]^ which serve as single‐molecule junctions for subsequent exploration of the intricate relationship between the physical and chemical properties of the molecule.^[^ ^177^, ^178^, ^179^, ^180^ ^]^

Nature harnesses mechanochemical transduction processes to accomplish a myriad of crucial functions, with a captivating example being the emission of light in response to mechanical stimuli.^[^ ^181^, ^182^ ^]^ However, effecting the conversion of force signals into luminescence at the molecular level poses a significant challenge. Notably, Rint P. Sijbesma's group has made progress in this area by demonstrating that bis‐adamantyl‐1,2‐dioxetane exhibits visible light emission when subjected to mechanical force either along the polymer chain or within networks containing the dioxetane unit^[^ ^59^, ^172^ ^]^ (Figure 5e). The light emission stems from the adamantane ketone excited state, which is formed upon the opening of the four‐membered dioxane ring.^[^ ^183^, ^184^ ^]^ The authors further refined this approach by enhancing the sensitivity and enabling color tuning through the utilization of energy generated from the cleavage of the four‐membered dioxygenated ring bond. This energy is leveraged to interact with various receptors, thereby facilitating the production of distinct fluorescence signals and augmenting light intensity. The exceptional spatial and temporal resolution afforded by this technique underscores its potential for probing the failure mechanisms of polymeric materials in unparalleled detail.

#### 3.2.2 Synergistic Coupling of Different Mechanophores

In addition, as depicted in Figure 5, certain ring cleavage reactions are not only mechanically reversible (e.g., rhodamine), but also reversible when subject heating (e.g., spiropyran), illuminating (e.g., bisthienylethene,) or chemical stimuli (e.g., lactone or lactam).^[^ ^173^, ^174^, ^185^, ^186^, ^187^ ^]^ This leads to coupled interactions that can occur in the same mechanophore, allowing for the simultaneous detection of mechanical and external chemical environmental changes. Chen's team utilized bisrhodamine (Rh) and bisthienylethene (BTE) to form a novel mechanophore molecule.^[^ ^187^ ^]^ Poly(methyl acrylate) containing this dual mechanical carrier can be mechanically activated by ultrasound. The relative distribution of the Rh ring‐opening products of two different colors and fluorescence varies with the magnitude of the force. Orthogonal utilization of the photochromic reaction of the BTE core enhances the mechanochromism of the polymer and gates the polymer's mechanofluorescence.

Force‐triggered mechanochemical reactions can induce measurable changes in macroscopic mechanical properties of materials, however, force‐triggered change of chemical properties in materials have not yet received attention. Stephen L. Craig's team reports on a double‐network hydrogel in which they have fused a 2‐ methoxy‐gem‐dichlorocyclopropane mechanoacid with a methyl methoxycyclobutene carboxylate mechanophore structure fused to form a gated structure.^[^ ^188^ ^]^ This gated mechanoacid was incorporated into linear and network polymers via radical copolymerization, which increased its internal solution acidity by an order of magnitude during stretching. Furthermore, after large deformations, the material recovers its initial shape when the external force is withdrawn. Sonication experiments confirmed the mechanical release of HCI, and single‐molecule force spectroscopy revealed enhanced single‐molecule toughness of the covalent chains. This composite mechanochemical functionality was integrated into a double‐network hydrogel, resulting in mechanically strong, thermally stable materials with strain‐triggered acid release.

### 3.3 Lever‐Arm Effects

Mechanical force serves as a direct modifier of the energy landscape, facilitating the activation of reaction pathways for mechanophores integrated within polymer chains.^[^ ^189^ ^]^ In addition, the mechanical response of mechanophores can be influenced by the structural characteristics of the polymer backbone responsible for imparting forces. This phenomenon stands in contrast to the dynamics of a tug‐of‐war game, wherein victory is typically determined by the properties of the participating team members rather than the properties of the rope itself. Mechanophores in polymers, much like knots in ropes, adjust the mechanics by altering the chain's structure.

**Figure 6:**
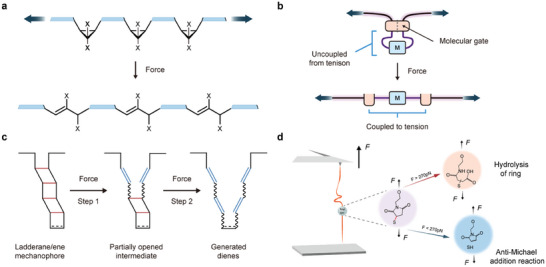
Lever‐arm effects and complex effects of polymer mechanochemistry. a) The gDCC force stretching causes unfolding to occur through positive feedback via Lever‐arm effects. Reproduced with permission.^[^ ^189^ ^]^ Copyright 2012, Springer Nature Limited. b) Cascade reactions due to molecular gate. Reproduced with permission.^[^ ^202^ ^]^ Copyright 2016, CC‐BY‐4.0. c) Cascade unzipping of ladderane. Reproduced with permission.^[^ ^204^ ^]^ Copyright 2020, Springer Nature Limited. d) Maleimide–thiol adducts stabilized through stretching. Reproduced with permission.^[^ ^207^ ^]^ Copyright 2019, Springer Nature Limited.

In mechanochemistry, the polymer framework serves a dual role: it acts not only as a “tether,” facilitating the transfer of force to the reactants but also as a “lever,” enhancing mechanochemical coupling efficiency.^[^ ^189^, ^190^, ^191^, ^192^, ^193^ ^]^ For example, in studies utilizing single molecular force spectroscopy, the forces associated with the ring opening of gem‐dibromo and gem‐dichlorocyclopropanes (gDHC) anchored along the backbone of *cis*‐polynorbornene and *cis*‐polybutadiene were directly quantified and compared.^[^ ^194^, ^195^ ^]^ Remarkably, the critical isomerization force within the polynorbornene scaffold was found to be approximately one‐third lower than that observed for polybutadiene, indicating that the polynorbornene backbone can accelerate the reaction rate relative to the polybutadiene backbone. Notably, the partitioning of extrinsically applied force among non‐reactive (i.e., primarily backbone) molecular degrees of freedom and the attendant kinetic and thermodynamic changes are more complex than presented in this section.^[^ ^196^, ^197^, ^198^ ^]^ Similarly, other single molecular force spectroscopy studies have elucidated the sensitivity of force‐induced acceleration in the electrocyclic ring opening of gem‐dichlorocyclopropanes (gDCCs) to the stereochemistry of an α‐alkene substituent on the gDCC molecule^[^ ^189^, ^199^, ^200^ ^]^ (**Figure** **6**a). These findings underscore the potential of similar backbone effects to be more effective than conventional structural modifications employed to facilitate force‐free reactions.

### 3.4 Complication Effects

In addition to the mechanisms described above, there exist additional intricate effects in polymer mechanochemistry, one of which is the influence of force‐induced steric hindrance. Force clamp AFM‐based SMFS experiments have demonstrated that the reduction of disulfide bonds in proteins results in an abrupt switch in reactivity at a critical force of ≈0.5 nN^[^ ^201^ ^]^ The underlying rationale for this intriguing observation can be attributed to the subtle interplay between two antagonistic effects induced by mechanical force. Typically, mechanical force works along the mechanical coordinate, thereby accelerating the reaction rate. Once a critical force threshold is reached, a specific distortion of the S–S–C–C dihedral angle of the disulfide moiety occurs. This rearrangement results in a conformer that is less susceptible to attack by a nucleophile due to steric hindrance.

Mechanochemistry, although primarily focused on individual reactions, also holds significant potential for complex reaction systems and feedback loops facilitated by cascade effects^[^ ^202^ ^]^ (Figure 6b). Similarly, this synergy of complex systems and cascading effects should be at work in living organisms.^[^ ^203^ ^]^ A prime example is the utilization of the mechanophore cyclobutane as a gate to regulate the activation of another mechanophore, dichlorocyclopropane.^[^ ^202^ ^]^ Experimental findings revealed that an additional force of 0.5 nN is necessary to mechanically isomerize a gated dichlorocyclopropane within a polymer compared to isomerizing free dichlorocyclopropane at an equivalent rate. This gating concept presents a promising mechanism for modulating stress‐responsive behaviors such as load strengthening and mechanical chromism. The mechanochemical cascade unzipping of ladderane demonstrates this phenomenon. Experimental observations have consistently shown that [4]‐ladderane exhibits “all‐or‐none” cascade mechanoactivation across various polymer backbones, resulting in the same stereochemical distribution of generated dienes. These effects are governed in solution environments by unique non‐equilibrium dynamic mechanisms: energy transduction from the first cycloreversion significantly accelerates the second cycloreversion, and bifurcation on the force‐modified potential energy surface dictates the product distributions. However, this effect is not well studied in the condensed phase. This elucidation marks a groundbreaking advancement in understanding the coupling effects between cascade mechanochemical reactions and the control of product ratios through kinetic bifurcations, thus significantly deepening our knowledge of mechanochemical mechanisms^[^ ^204^ ^]^ (Figure 6c). Furthermore, this research highlights the possibility of constructing novel materials previously deemed inaccessible through the chemical conversion of precursor macromolecules induced by mechanical transduction.

Traditionally, the coupling product of sulfhydryl and maleimide is unstable in vivo and prone to dissociation or thiol exchange reactions with molecules such as glutathione in the body, limiting its application in biomedicine.^[^ ^205^, ^206^ ^]^ Contrary to the anticipated mechanically induced chemical bond cleavage (Figure 6d), the coupling product promotes the hydrolysis of the maleimide ring. This results in the formation of a more stable hydrolyzed linker, thereby inhibiting the dissociation of sulfhydryl groups or the occurrence of sulfhydryl exchange reactions.^[^ ^207^ ^]^ This discovery has been leveraged to prepare antibody‐polyethylene glycol complexes whose stability in aqueous environments can be significantly enhanced through simple ultrasound treatment, underscoring the promising application prospects of this technology. The directionality of force plays a pivotal role in regulating reaction products by altering the dynamic distribution of reaction pathways in various directions under force. This directional control mechanism has been investigated extensively, offering insights into the manipulation of reaction outcomes through force modulation.^[^ ^59^, ^103^, ^205^, ^208^, ^209^ ^]^

### 3.5 Mechanical Responses of Polymer Materials

Instead of solely focusing on conventional processing techniques and macroscopic mechanical phenomena in material utilization, there has been a notable transition toward understanding mechanisms at the molecular level and elucidating the complex mechanical transformations induced by mechanical force. This shift underscores the realization that employed mechanophores into polymers can trigger nondestructive chemistry and intricate mechanical responses in materials, extending beyond simple bond scission and mechanical failure. By delving into the molecular intricacies and mechanistic underpinnings, researchers are better equipped to harness the full potential of mechanochemical processes for tailored material design and functionalization.

In the realm of materials design, the exploration of novel mechanochemistry phenomena has unveiled intriguing insights, including the distinct conformations adopted by organic molecules containing azobenzene when subjected to external forces. These isomer conversions result in markedly different physicochemical properties, paving the way for the development of a diverse array of emerging materials. For instance, proteins exhibit remarkably intricate and varied force chemistry patterns, providing a rich source of inspiration for fabricating toughened, stretch‐resistant, and fatigue‐resistant network structures. Additionally, these complex force chemistry patterns enable the precise orientation and protection of reactive groups within materials, further enhancing their utility and functionality. The widely used mechanophores include weak covalent bonds, coordination bonds, pericyclic reaction products and cycloaddition reaction products.^[^ ^9^, ^176^, ^210^, ^211^, ^212^, ^213^, ^214^, ^215^, ^216^, ^217^, ^218^, ^219^, ^220^ ^]^

It is well known that polymers are in a highly folded state within the material due to entropy, resulting in a large of bond energy releasing when stretching.^[^ ^221^, ^222^, ^223^, ^224^ ^]^ When a cyclic structure is present in the mechanophore, the breaking of specific chemical bonds allows for an increase in the length of the polymer profile. This significantly broadens the applications of polymers for stimuli‐responsive materials.^[^ ^183^, ^225^, ^226^, ^227^, ^228^, ^229^ ^]^

**Figure 7:**
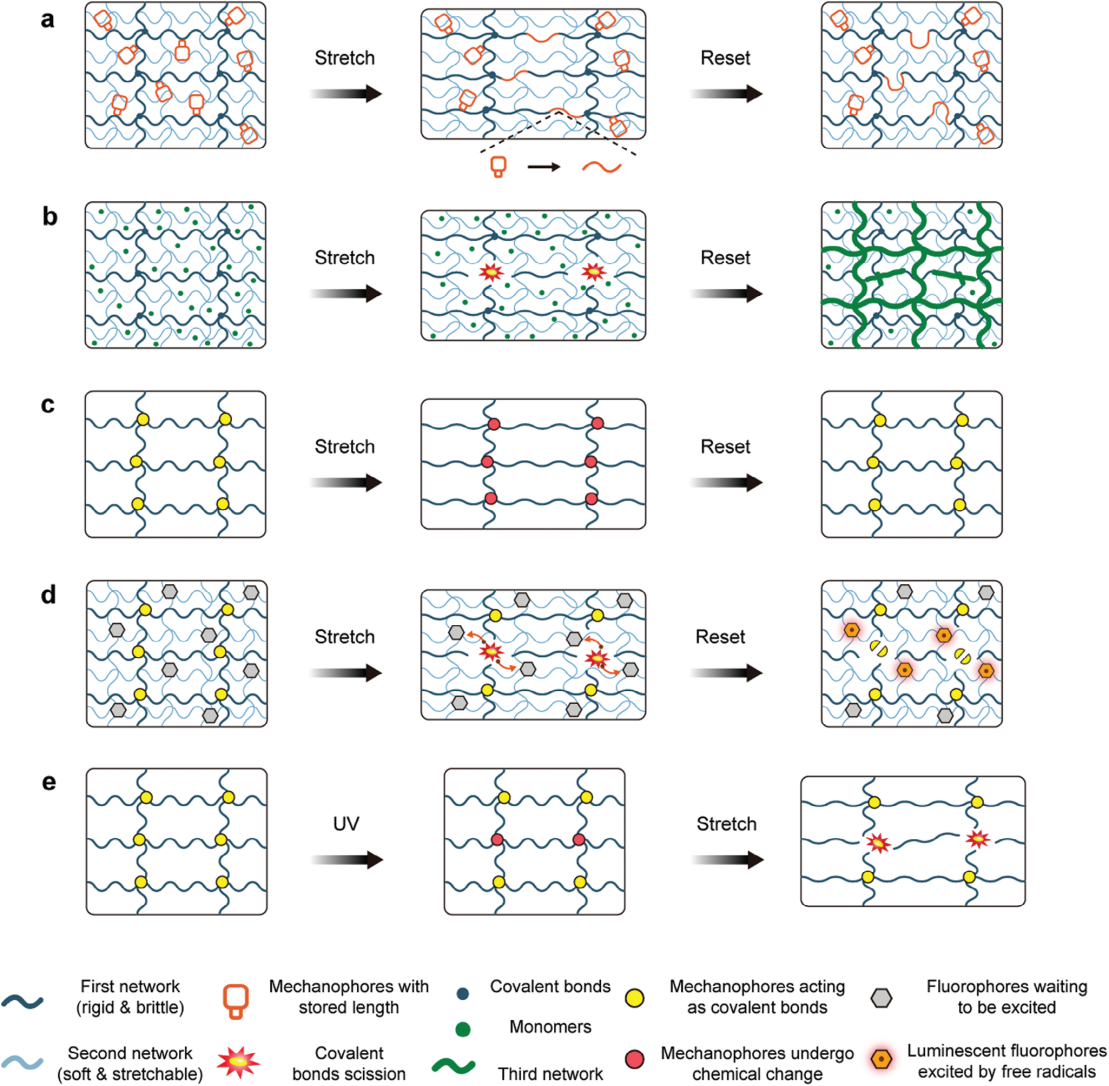
Macroscopic regulation of material properties through mechanophores. a) Covalent bond breaking within a mechanophore to generate new profile lengths. b) Network breaking to generate free radicals to promote localized or overall mechanical strength of the material. c–e) Mechanical properties are related to chemical properties through mechanical forces, and a mechanophore can be subjected to either a conformational change that results in a change in the macroscopic properties of the material (c) or the destruction of the mechanical structure generates free radicals thus inducing chemical reactions to occurred (d). On the other hand, a conformational change through a change in the external environment. isomer conversions that affect the mechanical properties (e).

The remarkable mechanochemical responses reported in the literature include coloration, luminescence, cross‐linking, depolymerization, small molecule release, catalysis, and mechanically gated activation, as shown in **Figure** **7**. For instance, Gong's team, pioneered the development of polymer networks wherein constituent chains undergo force‐coupled reactions when reaching their nominal breaking point. Notably, these stretched hydrogels exhibit twice the tearing energy compared to networks formed from similar control chains^[^ ^218^ ^]^ (Figure 7a). The breaking of covalent bonds within the cyclic molecule (depicted in red) not only releases energy but also increases the profile length of the polymer chain, consequently enhancing ductility. These enhancements complement the benefits of double network (DN) architecture and supplement existing strengthening strategies for polymer materials.^[^ ^230^, ^231^, ^232^ ^]^ There are also a number of ways to enhance the strength of the network, which include doping tough structures;^[^ ^233^, ^234^ ^]^ using hydrogen bonding to form internal structures;^[^ ^52^, ^234^, ^235^, ^236^ ^]^ realizing physical entanglement;^[^ ^237^, ^238^, ^239^ ^]^ forming microscopic topologies by phase separation;^[^ ^235^, ^240^, ^241^ ^]^ and building macroscopic topologies by braiding.^[^ ^230^, ^242^, ^243^ ^]^

Moreover, leveraging mechanochemistry for the development of new polymer materials and manufacturing techniques extends beyond traditional applications such as high‐toughness elastomer preparation, self‐healing materials, and force‐induced degradation polymer materials.^[^ ^244^, ^245^ ^]^ In polymer networks, the presence of activation energy leads to functional groups that require external stimuli before chemical reactions are possible, and mechanical response has gradually gained attention due to its simplicity and efficiency. Gong and colleagues introduced an imprinting method for growing microstructures on hydrogel surfaces, employing a force‐triggered polymerization mechanism within double‐network hydrogel, which enables rapid spatial modulation of hydrogel surface morphology and chemistry within seconds, offering on‐demand functionality.^[^ ^217^ ^]^ Additionally, they devised self‐growing materials based on the mechanical training of DN gels. Under mechanical stress, the brittle network (depicted in black) fractures, while the highly stretchable network (light blue) maintains gel integrity. Mechanical free radicals generated at the ends of brittle network chains trigger the polymerization of monomers from the external environment, leading to the formation of a new network (dark green). This design facilitates the mechanical training of flexible materials by initiating the generation of free radicals through mechanical stimulation, thereby leading to an additional reaction^[^ ^216^ ^]^ (Figure 7b).

Indeed, mechanical stimulation has the remarkable ability to induce isomer transformation reactions of chemical molecules, leading to significant alterations in their chemical properties.^[^ ^177^, ^212^, ^246^, ^247^, ^248^, ^249^, ^250^ ^]^ Nancy R‐Sotos et al. have capitalized on this phenomenon by exploiting the color‐changing property of spiropyrans in response to structural changes triggered by mechanical stimuli, which enabling the detection and mapping of mechanical stresses within monolithic polymer materials. This approach provides opportunities for the evaluation, modification, and improvement of materials prior to catastrophic failure^[^ ^9^ ^]^ (Figure 7c). This phenomenon highlights the dynamic nature of mechanochemistry and its potential applications in sensing mechanical forces within materials.^[^ ^251^ ^]^ Chen's team prepares an elastomer that uses diselenoids (Se‐Se) as the mechanophore to direct the disruptive force to bonding reactions^[^ ^252^ ^]^ (Figure 7d). Polyurethanes have been functionalized with labile selenium–selenium bonds, whose mechanical activation generates selenium radicals that trigger radical transfer and in situ cross‐linking reactions. The resulting covalent network has turned‐on mechanical fluorescence and increased modulus, providing stress reporting, mechanical healing, and mechanical remodeling of the deformed membrane.

Equally significant as mechanically regulated changes in chemical molecules are isomer conversions that can influence the mechanochemistry of materials containing those molecules.^[^ ^188^, ^253^, ^254^, ^255^ ^]^ Li and collaborators demonstrated that photoinduced structural changes in azobenzene can alter its breaking force, rendering it an ideal photoresponsive mechanical carrier.^[^ ^214^ ^]^ Mechanophore A (depicted in yellow) undergoes a cis‐trans isomerization change upon UV irradiation to form mechanophore B (depicted in red), leading to a change in bonding energy and consequently altering the fracture energy of the material (Figure 7e). These mechanical characteristics of azobenzene offer a means to rationally manipulate the macroscopic fracture behavior of polymer networks through light irradiation. Leveraging light‐induced isomer transformations to modify the mechanical response of functional groups is an appealing approach for designing polymer networks with photoadjustable mechanical properties. This highlights the potential of utilizing light‐induced isomer conversions to tailor the mechanical properties of polymers for various applications.

## 4 Interface Mechanochemistry

Mechanical force also plays a significant role in altering the structure and physical–chemical properties of interfaces, forming the cornerstone of interface mechanochemistry. While one might initially associate interface mechanochemistry with actions such as drilling wood to initiate a fire, thereby activating chemical reactions through frictional force, it is important to note that tribological mechanochemistry is beyond the scope of this discussion. Interested readers are referred to recent comprehensive reviews^[^ ^256^, ^257^, ^258^, ^259^, ^260^ ^]^ on this topic.

In the realm of materials science, particularly in fields such as composite materials, the interface often serves as a focal point due to its role as a stress concentration area. Consequently, the mechanical properties of composites are frequently dictated by the characteristics of their interfaces. Early endeavors in interface mechanochemistry were primarily concentrated on exploring the behavior of metal alloys.^[^ ^257^, ^261^ ^]^ Within metal alloys, lattice distortions, electron rearrangements, and defect recombination processes are intricately governed by interface stress and external mechanical forces.^[^ ^255^ ^]^ In solid fuel cells, where the migration of ions within electrode materials is influenced by stress, interfacial mechanochemistry plays a crucial role in ensuring mechanical stability and safety.^[^ ^262^, ^263^, ^264^ ^]^ Expanding beyond traditional materials science, interfaces also hold immense importance within biological systems. For instance, interfaces between tissues and bones in the human body are ubiquitous. Understanding the mechanical properties of these biological interfaces and developing biomimetic materials for various applications have emerged as captivating areas within interface mechanochemistry.^[^ ^265^, ^266^, ^267^, ^268^, ^269^, ^270^, ^271^ ^]^

### 4.1 Polymer‐Based Interfacial Adhesion

#### 4.1.1 Conventional Modes of Polymer‐Based Interfacial Adhesion

Polymers are extensively utilized in various aspects of daily life owing to their remarkable processability, compatibility for doping with diverse substances, and facile chemical modification. The utility of these materials in interfacial adhesion has garnered significant attention from researchers, as evidenced by numerous studies in the literature.^[^ ^272^, ^273^, ^274^, ^275^, ^276^ ^]^ This section specifically describes the interfacial adhesion properties of hydrogel materials derived from synthetic polymers.

Hydrogels stand out in novel materials due to their unique softness, wetting properties, reactivity, biocompatibility, and biological activity. However, achieving robust adhesion akin to that observed in soft connective tissues such as tendons, ligaments, and cartilages to bones, which can sustain high toughness (≈800 J m^−2^) over millions of mechanical loading cycles, poses a significant challenge in synthetic hydrogels and engineering materials. Nonetheless, such fatigue‐resistant adhesion is highly coveted for a myriad of applications, including artificial cartilage and tendons, resilient antifouling coatings, and hydrogel robots. Studies have revealed that the adhesion energy comprises a combination of interfacial adhesion energy and energy dissipated by gel extension. Hence, the mechanical properties of the adherent material are just as critical as its adhesion capacity, with mechanophores serving as intrinsic mechanical structures capable of exerting significant influence.

**Figure 8:**
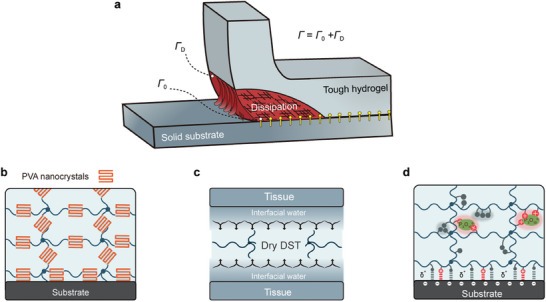
Examples of polymer‐based interfacial adhesion. a) Components of adhesion energy. Reproduced with permission.^[^ ^277^ ^]^ Copyright 2016, Springer Nature Limited. b) Ordered nanostructures acting as adhesion mechanophores through hydrogen bonding. Reproduced with permission.^[^ ^281^ ^]^ Copyright 2020, CC‐BY‐4.0. c) The hydrophobic groups unfold the structure to expel the remaining water molecules at the interface, contributing to adhesion formation. Reproduced with permission.^[^ ^282^ ^]^ Copyright 2019, Springer Nature Limited. d) Polar molecular polymers form adhesive structures. Reproduced with permission.^[^ ^283^ ^]^ Copyright 2022, ACS Publication.

Surface adhesion arises from a complex interplay of physical interactions and intermolecular forces, encompassing hydrogen bonds, electrostatic interactions, van der Waals forces, and covalent bonds. In the field of hydrogel‐based adhesives, various design strategies have been developed. For example, Zhao lab reported a strategy to design synthetic hydrogels that adhere to various solids through chemical anchoring^[^ ^277^ ^]^ (**Figure** **8**a). This was achieved by cross‐linking long‐chain polymer networks to functional silanes grafted onto the surfaces of various solids, leading to interfacial toughness values exceeding 1000 J m^−2^. However, such robust hydrogel adhesion is susceptible to fatigue failure over multiple cycles of mechanical loading, wherein the effect of bulk dissipation becomes depleted. In response to this challenge, a bioinspired strategy has been proposed to achieve fatigue‐resistant adhesion by anchoring ordered nanostructures onto engineering materials.^[^ ^224^, ^278^, ^279^, ^280^ ^]^ This approach capitalizes on the observation that compared with amorphous polymer chains, ordered nanostructures require significantly more energy for fatigue‐crack propagation^[^ ^281^ ^]^ (Figure 8b).

Indeed, achieving adhesion on wet surfaces, such as body tissues, poses significant challenges due to the ability of water to separate molecules from contact surfaces, thereby impeding interactions.^[^ ^284^, ^285^, ^286^ ^]^ Current adhesives for wet adhesion such as tissues, typically in the form of liquids, primarily function through the diffusion of their molecules across interfacial water to bond with the polymer networks of tissues. However, they suffer from several limitations: weak bonding, low biological compatibility, poor mechanical match with tissues, and slow adhesion formation.^[^ ^287^, ^288^, ^289^, ^290^ ^]^ Surprisingly, a recent breakthrough involves the development of a novel alternative tissue adhesive in the form of dry double‐sided tape (DST), composed of a combination of a biopolymer (gelatin or chitosan) and crosslinked poly(acrylic acid) grafted with N‐hydrosuccinimide ester^[^ ^282^ ^]^ (Figure 8c). The adhesion mechanism of this DST relies on the removal of interfacial water from the tissue surface, facilitating rapid temporary crosslinking to the surface. Subsequent covalent crosslinking with amine groups on the tissue surface further enhances the adhesion stability and strength of the DSTs. This pioneering work opens up exciting new avenues for applications in bioscaffolds, drug delivery, and wearable and implantable devices.

Due to the electrical interactions at the interface, the enrichment of polar molecules has emerged as a promising approach to enhancing adhesion.^[^ ^291^ ^]^ However, the inherent mutual repulsion among polar molecules presents a challenge in maintaining a high concentration at the interface to achieve optimal adhesion. To address this challenge, Meng's research group has developed a gel material that demonstrates strong adhesion and high toughness by leveraging the synergistic effects of electrostatic and hydrophobic interactions. These hydrogels exhibit remarkable adhesion strength and exceptional tensile properties and serve as an ideal model system for investigating the mechanisms underlying strong adhesion in hydrophobic hydrogels and broadening the application scope of hydrogels^[^ ^283^ ^]^ (Figure 8d). This approach is also applied in the preparation of bionic adhesive materials, which will be presented in Section 4.2.

#### 4.1.2 Polymer Topological Interfacial Adhesion

**Figure 9:**
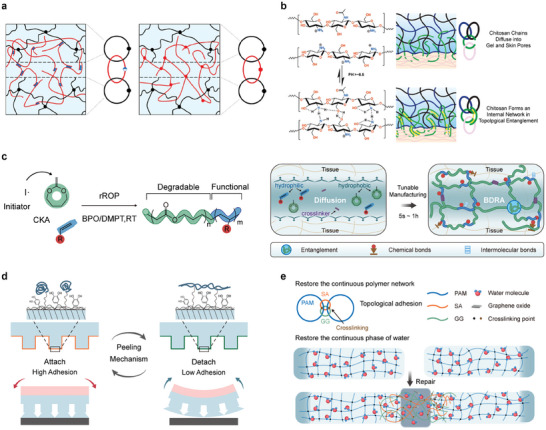
Principles and applications of topological adhesion. a) Conventional adhesion (left) compared with topological adhesion (right). Reproduced with permission.^[^ ^306^ ^]^ Copyright 2018, Wiley. b) Strong adhesion without covalent bonding is produced by the presence of chitosan chains across the interface. Reproduced with permission.^[^ ^308^ ^]^ Copyright 2022, Wiley. c) Topological adhesion can be transformed into covalent bonding, which strengthens the original network and its adhesion capacity. Reproduced with permission.^[^ ^309^ ^]^ Copyright 2023, Springer Nature Limited. d) The topological adhesion capacity is related to the structure of the material, which is utilized to prepare interfaces with tunable adhesion capacities. Reproduced with permission.^[^ ^312^ ^]^ Copyright 2024, Springer Nature Limited and Reproduced with permission.^[^ ^313^ ^]^ Copyright 2021, ACS Publication. e) Rapid self‐healing of single materials by topological adhesion. Reproduced with permission.^[^ ^317^ ^]^ Copyright 2024, AAAS.

Polymer mechanophores, with their macroscopically flexible yet microscopically rigid structure, facilitate the formation of catenane, rotaxane and other mechanically interlocking structures.^[^ ^292^, ^293^, ^294^, ^295^, ^296^, ^297^, ^298^ ^]^ These structures form mechanical bonds through topological relationships to enhance their macroscopic mechanical properties.^[^ ^299^, ^300^, ^301^, ^302^ ^]^ Suo's group introduced the concept of topological adhesion, which involves the formation of a crosslinked layer through the interpenetration of polymer chains. This adhesion arises from the entanglement or site‐blocking effects of the two components, even in the absence of covalent interactions.^[^ ^256^, ^303^, ^304^, ^305^, ^306^, ^307^ ^]^ This innovative approach has led to the development of a plethora of adhesive materials with applications ranging from wearable flexible electronics to underwater adhesives and surgical adhesives^[^ ^305^, ^306^ ^]^ (**Figure** **9**a). Moreover, David J. Mooney's team pioneered the creation of a tough adhesive (TA) by combining pH‐responsive bridging chitosan polymer chains with a tough dissipative hydrogel matrix. The adhesion mechanism primarily relies on the topological entanglement between the chitosan chains and the permeable adhered surface^[^ ^308^ ^]^ (Figure 9b).

Adhesives possessing both strong adhesion and tunable degradability are indispensable in clinical and ecological contexts, yet their fabrication presents a significant challenge. Luan et al. introduced an innovative approach for designing a robust adhesive with a degradable backbone suitable for physiological environments (BDRA) using an in situ radical ring‐opening polymerization (rROP) strategy^[^ ^309^ ^]^ (Figure 9c). Subsequently, redox‐initiated in situ rROP forms a deep covalent interpenetrating network with a degradable backbone, offering a straightforward and environmentally friendly engineering approach.

Current adhesive technologies face diverse limitations that require urgent resolution, with a key concern being secondary damage caused during removal.^[^ ^310^, ^311^ ^]^ Previous studies have mimicked the adhesion properties of geckos and octopuses and have been inspired by the adhesion mechanism of mussels to construct microstructures on surfaces that can achieve underwater adhesion.^[^ ^285^ ^]^ Recently, Wang's team^[^ ^312^ ^]^ and Zhou's team^[^ ^313^ ^]^ used temperature‐responsive polymers that undergo conformational changes with temperature, achieving microscopic or macroscopic structural changes to enable reversible adhesion (Figure 9d). This method is expected to broaden the application range of hydrogels in various fields, such as tissue adhesives, wound sealants, wearable devices, and hydrogel‐metal protection.

The restoration of weight‐bearing and weight‐sensing capabilities in hydrogel materials following injury represents a captivating yet underexplored domain.^[^ ^314^, ^315^, ^316^ ^]^ Traditional repair techniques that rely on hydrogen bonding or van der Waals forces tend to be less effective due to the high water content of hydrogels. In response, Tang and colleagues devised a novel composite mucoadhesive gel (SGG) comprising sodium alginate, guar gum, and graphene oxide for repairing damaged hydrogels^[^ ^317^ ^]^ (Figure 9e). In addition, they developed an optimized repair method incorporating a cross‐shaped sectional (CSS) enhancement strategy to restore the loading and sensing capabilities of the hydrogel. The components of the SGG bonding agent interact with the damaged hydrogel in all orientations, resulting in complex entanglement with the polymer network near the cracks. The SGG adhesive infiltrates the hydrogel, forming an intermediate gel layer, and subsequently cures to repair the material through topological adhesion. This process restores the continuity of the polymer network and the aqueous phase within the hydrogel. Moreover, the strain capacity is significantly restored, providing an effective approach for rehabilitating wearable devices.

#### 4.1.3 Biologically Inspired Novel Polymers for Interfacial Adhesion

Adhesion in aqueous solutions poses significant challenges due to the isolating effect of water molecules. In recent years, biomimetic materials have garnered increasing attention as a research focus. Natural organisms such as mussels, sandcastle worms, and barnacles exhibit remarkable underwater adhesion abilities. DOPA and its derivatives, which are secreted by mussels, are particularly noteworthy for their strong adhesion effect attributed to their benzene ring structure, which facilitates cation‐π interactions with cationic amino acids.^[^ ^318^ ^]^ Since not all parts of the original protein have the ability to adhere and it is difficult to obtain them, the selection of functional groups in DOPA for the preparation of polymeric materials to achieve stronger adhesion is a popular research direction nowadays.^[^ ^319^, ^320^, ^321^, ^322^, ^323^, ^324^, ^325^, ^326^, ^327^, ^328^ ^]^ Similarly, sandcastle worms secrete a solution containing positive and negative ions, leading to adhesion through robust electrostatic interactions. Inspired by these natural adhesive mechanisms, Wang's team developed a bionic natural binder by combining positively charged quaternized chitosan with negatively charged sodium alginate. This innovative approach enables strong bonding between various types of solid particles, such as sand and slag, ultimately yielding high‐strength, low‐carbon building materials under low‐temperature, normal‐pressure conditions.^[^ ^329^ ^]^

### 4.2 Natural Molecule‐Based Interfacial Adhesion

Another strategy in the design of interface adhesives involves incorporating natural adhesion molecules such as the adhesive proteins found in mussels and barnacles.^[^ ^330^ ^]^ The application of surface force apparatus (SFA) and atomic force microscopy (AFM) technologies in interface mechanochemistry has facilitated a deeper understanding of interfacial adhesion at the molecular level.^[^ ^331^ ^]^ Adhesive proteins serve as exemplary models in this context, shedding light on the intricate relationships among protein sequence, structure, and adhesion strength.

#### 4.2.1 Adhesion of Mussel Proteins and Their Derivatives

**Figure 10:**
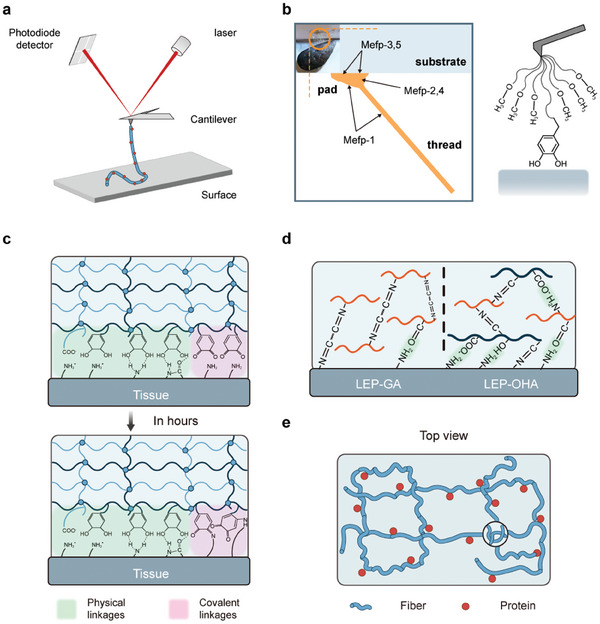
Natural molecule‐based adhesion. a) Schematic diagram of single‐molecule dissociation for mechanical property testing of proteins. b) Single‐molecule dissociation studies of the Mfps structural domain to search for natural adhesion protein fragments. c) Preparation of event‐related adhesion gels based on DOPA and its derivatives. Reproduced with permission.^[^ ^346^ ^]^ Copyright 2021, Springer Nature Limited. d) Injectionable adhesion gels prepared by repetitive amino acid interactions with organisms. Reproduced with permission.^[^ ^348^ ^]^ Copyright 2022, ACS Publication. e) Amyloid and elastin self‐assembled fibers have an extremely strong adhesion capacity; this picture shows the top view. Reproduced with permission.^[^ ^351^ ^]^ Copyright 2024, Wiley.

Early investigations have elucidated the pivotal role of the unique amino acid 3,4‐dihydroxy‐L‐phenylalanine (DOPA) in the adhesive properties of mussel foot proteins (Mfps), facilitating robust attachment to diverse surfaces under aqueous conditions. Notably, bulk surface force apparatus (SFA) experiments have underscored the necessity of a high adhesion energy, approximately on the order of ≈3×10^−4^ J m^−2^,^[^ ^332^, ^333^ ^]^ for the separation of two mica surfaces bridged by Mfp‐3. Subsequent studies delved into the mechanistic underpinnings of this robust adhesion (**Figure** **10**a). A significant number of positively charged residues, such as lysine (Lys) and arginine (Arg), are juxtaposed with DOPA within Mfps. Binding experiments utilizing small‐molecule cyclic synthetic analogs, with Lys (or Arg) adjacent to catechol (or phenyl) groups, to wet mica have demonstrated remarkably elevated adhesion energies ≈15×10^−3^ J m^−2^,^[^ ^334^, ^335^ ^]^ suggesting an adaptive synergy between motifs featuring adjacent catechol‐lysine pairs. Conversely, recent SFA measurements on peptides containing phenylalanine (Phe) and Lys have yielded contrasting results, wherein DOPA‐deficient Mfps exhibit even stronger wet adhesion capabilities than their DOPA‐containing counterparts. This enhanced adhesion has been primarily attributed to the interaction of Lys with the mica surface and intermolecular cation‐π interactions between Lys and Phe residues.

To gain a comprehensive understanding of the mechanochemical mechanisms underlying the strong adhesion of mussel proteins at the single‐molecule level, a diverse array of SMFS experiments have been conducted.^[^ ^332^, ^333^, ^336^, ^337^, ^338^, ^339^ ^]^ A seminal study by Li et al. employed a “multi‐fishhook” approach to investigate the adaptive binding of DOPA to various wet surfaces, yielding a range of 60–90 pN with a protected Lys side chain.^[^ ^340^, ^341^, ^342^ ^]^ Notably, a significantly greater detachment force of 300 pN was observed between DOPA and the mica surface when the Lys dipeptide was involved (Figure 10b). Additionally, Li and colleagues explored the single‐molecule adhesive behavior of Mfp‐5, revealing that parameters such as binding site density, peptide length, and topology significantly influence adhesion and cohesion properties.^[^ ^343^, ^344^ ^]^

In contrast to adhesive proteins found in mussels, the bacterial recombinant Balanus albicostatus CP19K (rBalCP19K) lacks DOPA or any other amino acids featuring post‐translational modifications. Nonetheless, it can self‐assemble into aggregated nanofibers under acidic pH conditions and demonstrates robust adhesion.^[^ ^345^ ^]^ These discoveries offer a novel approach for optimizing the synthesis of aqueous phase adhesives.

Hence, the utilization of DOPA and its derivatives in adhesive material design becomes imperative.^[^ ^285^, ^287^, ^334^ ^]^ However, the challenge lies in the propensity for side reactions, particularly oxidation, which is often difficult to control. Therefore, the stabilize ion and control of DOPA oxidation are pivotal. Xue et al. addressed this issue by employing electroformed dopaquinone oxide to fabricate adhesive materials, revealing time‐dependent adhesion between the hydrogel band and tissue surface^[^ ^346^ ^]^ (Figure 10c). Initially, primarily noncovalent interactions such as ionic interactions, cation‐π interactions, and hydrogen bonding are established within a few seconds. The application of this material in both in vitro organ models and in vivo animal studies demonstrated outstanding underwater adhesion and rapid hemostatic effects, thus introducing a novel research direction for adhesive material preparation.

#### 4.2.2 Adhesion of Amino Acid Repeat Sequences

In natural compounds, in addition to the recognized ability of lysine to adhere to DOPA, researchers have been intrigued by the potentially similar abilities of other amino acids.^[^ ^347^ ^]^ Qu's group addressed this curiosity by synthesizing P(MArg‐FHVI‐AA) hydrogels through the copolymerization of arginine derivative monomers, ionic liquid imidazolium salt derivative monomers, and acrylic acid (AA). Upon the introduction of metal ions, the mechanical and adhesive properties of the hydrogels were further enhanced while maintaining good biocompatibility and antimicrobial characteristics. Such materials hold promise as candidates for applications in smart wearable technology, health monitoring, and soft body materials^[^ ^348^ ^]^ (Figure 10d).

#### 4.2.3 Synergistic Adhesion of Amyloid to Elastin

Bioinspired design principles rooted in mimicking interfacial proteins have been widely employed, leading to the development of various catechol‐functionalized polymers suitable for biocompatible adhesives, self‐healing hydrogels, and materials for surgical wound closure. However, the limited availability of analogous methods for inducing desired globular proteins into peptide hydrogels has posed challenges to these designs.^[^ ^349^, ^350^ ^]^ It is imperative to integrate multiple macroscopic material properties into a single material, which is required for successful tissue engineering or biomedical applications. Chen's team elucidated an easily implementable and tunable self‐assembly strategy utilizing Ure2 amyloid peptides. This approach induces the assembly of any target protein into a supramolecular hydrogel, either individually or in combination, with significant control over the composition^[^ ^351^ ^]^ (Figure 10e). Moreover, this self‐assembled amyloid structure exhibits remarkably strong adhesion properties, offering a novel avenue for the preparation of protein‐mimetic materials in the future.

### 4.3 Mechanochemistry Printing

#### 4.3.1 Development of Precise Patterning Techniques for Surface Biomolecules

The precise patterning of surface biomolecules holds paramount significance across various domains, including proteomics research, drug screening processes, and population immunoassays. Traditional micromachining techniques, such as photolithography (PL), electron beam lithography (EBL), nanoimprint lithography (NIL), and chemical lift‐off lithography (CLL), have been widely employed to generate biomolecular patterns.^[^ ^352^, ^353^, ^354^, ^355^, ^356^ ^]^ However, these conventional methods are often considered “destructive”, as they necessitate the use of specific blocking agents and are typically incompatible with aqueous solutions.

**Figure 11:**
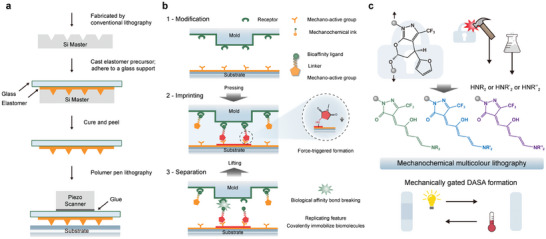
Development and application of mechanochemistry printing. a) Polymer Pen Lithography with Soft Tip. Reproduced with permission.^[^ ^358^ ^]^ Copyright 2008, AAAS. b) Principles of mechanochemical lithography. Reproduced with permission.^[^ ^379^ ^]^ Copyright 2022, ACS Publication. c) Principles and applications of spatiotemporal templated activation for mechanochemical multicolor printing. Reproduced with permission.^[^ ^380^ ^]^ Copyright 2023, Springer Nature Limited.

Several constructive strategies have been developed for producing patterned microstructures by directly delivering ink onto surfaces^[^ ^357^, ^358^ ^]^ (**Figure** **11**a). For example, Whitesides and colleagues introduced microcontact printing (µCP), which enables the deposition of various small molecules or proteins through physical or chemical adsorption.^[^ ^359^, ^360^, ^361^ ^]^ Gaub and collaborators devised a single‐molecule shear‐paste technique utilizing the tip of an AFM for transporting and depositing biomolecules.^[^ ^362^, ^363^, ^364^ ^]^ Additionally, Mirkin and team pioneered the application of dip‐pen nanolithography (DPN) and polymer pen lithography (PPL) for fabricating composite protein arrays.^[^ ^358^, ^365^, ^366^ ^]^ Despite their utility, these methods still encounter several limitations. These drawbacks include restricted ink loading capacity, viscous resistance hindering the diffusion of high‐molecular‐weight proteins, reduced protein bioactivity upon drying, and unstable attachment to the substrate.

#### 4.3.2 Development of Mechanochemistry Printing

Recently, there have been advancements in techniques that harness external energy to initiate chemical reactions at specific print locations. Such methods have the potential to enhance pattern resolution and prevent uncontrolled broadening of features during the liquid transfer process. Moreover, these techniques offer the possibility of establishing stable bonds between ink molecules and the surface through the formation of covalent bonds. For example, AFM tips coated with catalytic metals^[^ ^367^ ^]^ or enzymes^[^ ^368^ ^]^ have been employed to modify the local chemical composition of surfaces. Additionally, nanoscale tips capable of transmitting heat,^[^ ^369^, ^370^ ^]^ focusing light,^[^ ^371^, ^372^ ^]^ applying electric fields,^[^ ^373^, ^374^ ^]^ or inducing forces^[^ ^375^, ^376^ ^]^ have been utilized to trigger reactions on substrate surfaces, facilitating subsequent biomolecule patterning. However, it is worth noting that many of these methods cannot be performed continuously or directly in aqueous solutions. Furthermore, cutting‐edge nanofabrication techniques that remove surface material through mechanical reactions have been developed for fabricating ultrahigh‐resolution nanopatterns. These techniques represent promising avenues for achieving precise control of surface features at the nanoscale.^[^ ^377^, ^378^ ^]^

#### 4.3.3 Preparation of Mechanochemical Lithography using Bio‐Mechanophores

However, since the energy required for these mechanochemistry reactions is sufficient to destroy the protein structure, these methods are not directly applicable for printing biomolecules. Building upon insights from previous studies, Cao's team developed a novel approach termed mechanochemical lithography (MCL)^[^ ^379^ ^]^ (Figure 11b). The biophilic ligands aid in concentrating mechanochemical lithography initiators (MCIs) from the surrounding solution onto the molded surface. The resulting patterns were verified through fluorescence imaging. Furthermore, we demonstrated the ability to create multiple protein patterns using this technique. This innovative approach holds promise for precise and versatile biomolecule patterning applications.

#### 4.3.4 Erasable Mechanochemistry Printing

As a result, an array of chemistry lithography techniques has been developed and is gaining prominence. In pursuit of erasable lithography, Xu's team introduced the dynamic responsiveness of diselenides to establish a novel type of smart interface. Employing microcontact printing (uCP), the authors constructed diselenide patterns on quartz substrates. This approach facilitated a rapid patterning process that was dynamically erasable and compatible with various fluorescent molecules employed in the study.^[^ ^381^ ^]^ Inspired by these findings, Roman Boulatov and his collaborators Wengui Weng discovered that coumarin dimers are optically inert but undergo optical cleavage upon mechanical stimulation.^[^ ^382^ ^]^ This stimulus‐responsive gating of chemical reactions is highly intriguing and paves the way for the concurrent integration of mechanochemistry and photocatalysis to achieve erasable mechanochemical lithography. This innovative approach holds significant potential for advancing the field of lithography and enabling dynamic patterning processes with precise control.

#### 4.3.5 Mechanochemistry Multicolor PRINTING

The use of mechanically gated photoswitches, which become photosensitive only after mechanical forces remove the masking group, has significantly broadened the range of mechanical carriers based on well‐studied photoswitches.^[^ ^383^ ^]^ However, fine‐tuning the photophysical properties may require considerable synthetic effort, as each structural derivative might necessitate separate and often impractical synthetic routes. To address this challenge, Robb and colleagues introduced a novel technique termed spatiotemporal templated activation for mechanochemical multicolour printing (STAMMP), which expands the repertoire of mechanoswitchable photocarriers (Figure 11c).^[^ ^380^ ^]^ This method effectively extends the principles of soft lithography by designing an intricate mechanocarrier platform based on the scaffold of donor‐acceptor Steinhauser adducts (DASAs). By leveraging DASA‐based mechanocarriers, the authors demonstrated the feasibility of achieving mechanochemically controlled multicoloring in both solution and the solid state. This innovative approach holds promise for advancing the field of mechanochemical lithography and expanding its applications across different domains.

## 5 Biomechanochemistry

In contrast to the mechanochemistry elucidated previously, biomechanochemistry delves into the mechanisms by which cells respond to forces mediated by biomacromolecules, particularly proteins.^[^ ^384^, ^385^, ^386^ ^]^ The tertiary structure of biomacromolecules is intricately governed by various intramolecular interactions, including hydrogen bonds, ionic bonds, disulfide bonds, and hydrophobic interactions. These interactions, ranging from weak to strong, collectively shape the final 3‐D conformation of the biomacromolecule and dictate its functional attributes.

Undoubtedly, the influence of mechanical forces permeates living organisms. Numerous studies have provided evidence that cells can sense and respond to physical forces. For example, shear stress governs the diameter of blood vessels, muscle contraction dictates tissue stretching, and gastrointestinal motility generates low‐frequency vibrations. Additionally, cells actively probe their surroundings through physical forces, which are potent enough to induce the differentiation of mesenchymal stem cells, initiate transcriptional programs, drive morphogenesis, dictate cell migration, and regulate malignancy. In contemporary biomedical research, investigations into cancer cells and T cells have assumed increasing significance due to their involvement in various life‐threatening diseases. The mechanisms underlying these cellular responses to mechanical forces have traditionally been attributed to one‐step mechanochemical switches located at cell‐extracellular matrix (ECM) adhesions, cell‒cell adhesions, the plasma membrane, and the nucleus. These biological processes underscore the fundamental role of force regulation in molecular interactions within living organisms.

### 5.1 Mechanical Regulation of Molecular Processes

#### 5.1.1 Probing Protein Mechanical Structure and Mechanical Properties by SMFS

**Figure 12:**
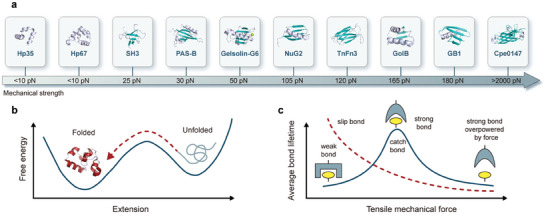
Mechanical regulation of protein molecular processes. a) Unfolding force of common protein molecules. The loading speed of the experiments was 0.4 µm s^−1^. b) Explanation of the folding–unfolding two‐state transition relationship of common protein molecules in a 1‐D free energy picture. Reproduced with permission.^[^ ^407^ ^]^ Copyright 2016, Springer Nature Limited. c) Catch bonds will have a longer key life for a given tension; however, slip bonds are monotonically shorter. Reproduced with permission.^[^ ^432^ ^]^ Copyright 2008, Annual Reviews.

Under mechanical force, proteins undergo changes in conformation, including unfolding, thereby converting the mechanical signal into a chemical signal that initiates downstream biochemical reactions. The mechanical unfolding of proteins has been extensively investigated since Gaub's group conducted a landmark study observing the reversible unfolding of individual titin immunoglobulin domains using AFM, which marked the inception of single‐molecule force spectroscopic technology.^[^ ^387^, ^388^, ^389^, ^390^, ^391^, ^392^ ^]^ AFM, count‐in magnetic tweezers and optical tweezers constitute the three most widely used techniques in single‐molecule force spectroscopy. **Figure** **12**a illustrates the unfolding forces of various proteins observed through SMFS experiments conducted in recent years, depending on certain pulling speed. The pulling speed of the experiment was 0.4 µm s^−1^.^[^ ^207^, ^340^, ^341^, ^342^, ^393^, ^394^, ^395^, ^396^, ^397^, ^398^, ^399^, ^400^, ^401^, ^402^, ^403^, ^404^, ^405^, ^406^ ^]^

The protein folding and unfolding kinetics are notably akin to diffusion over a 1‐D energy landscape, as convincingly demonstrated^[^ ^407^ ^]^ (Figure 12b), resembling the behavior observed in chemical reactions studied with SMFS. Therefore, it enables precise measurement and analysis of parameters such as the profile length, unfolding force, and interaction lifetime of proteins.^[^ ^404^, ^405^, ^408^, ^409^, ^410^, ^411^, ^412^, ^413^, ^414^, ^415^, ^416^, ^417^, ^418^ ^]^

The unfolding forces of proteins typically remain relatively low, often in the range of a few hundred piconewtons, owing to the presence of weak interactions such as hydrogen bonds, cation‐π interactions, and ionic bonds. Conversely, proteins also feature various covalent bonds, including disulfide bonds, single thiol–metal bonds, and isopeptide bonds, which play crucial roles in regulating protein structures and functions.^[^ ^402^, ^419^, ^420^ ^]^ However, it is noteworthy that the mechanical rupture forces of many covalent bonds deviate from those observed in conventional chemistry.^[^ ^421^, ^422^, ^423^, ^424^ ^]^ Such deviations in bond strength might be attributed to the influence of the microenvironment and the specific structural characteristics of the protein.

#### 5.1.2 Catch Bonds and their Mechanochemistry

Furthermore, SMFS has also been employed to investigate protein–protein interactions. Intriguingly, recent research has revealed the extraordinary mechanostability of a pathogen adhesion system. Specifically, the prototypical staphylococcal adhesion protein SdrG, which targets a short peptide from human fibrinogen β, forms a complex that can withstand forces of up to 2 nanonewtons—a magnitude previously associated with the strength of a covalent bond. The mechanism underlying this remarkable stability lies in the unique conformation of the target peptide, which is intricately confined in a screw‐like manner within the binding pocket of SdrG. This arrangement allows for the distribution of forces primarily toward the peptide backbone through a sophisticated hydrogen bond network.^[^ ^425^, ^426^, ^427^, ^428^, ^429^, ^430^ ^]^

Mechanical force typically weakens most protein–protein interactions. However, under specific conditions, applying an appropriate mechanical force can significantly enhance the material stability.^[^ ^431^ ^]^ This unique form of interaction, contrary to the convention of decreasing bond lifetime with increasing force, is termed a “Catch bond” (Figure 12c).^[^ ^432^ ^]^ Various explanations have been proposed for its origin, but the prevailing view suggests that the conformational entropy resulting from internal structural changes during directed stretching contributes to an overall free energy landscape characterized by extremely high potential barriers. Consequently, reaching a steady state requires overcoming these barriers.^[^ ^433^, ^434^, ^435^, ^436^, ^437^, ^438^ ^]^

Studies have revealed that the presence of a pin‐like structure at the end of the Fgb protein undergoes a conformational change during forward abstraction from the N2 and N3 domains of the SdrG protein. This conformational change prompts the pin‐like structure to traverse the entire protein, significantly extending both the bond energy and bond lifetime. Consequently, these adhesins can attach to their target with exceptional mechanostability, which is largely independent of peptide side chains.^[^ ^427^, ^428^ ^]^ This interaction represents the strongest mechanical noncovalent protein–protein receptor–ligand interaction observed to date and is capable of unfolding virtually any protein.^[^ ^403^ ^]^

In numerous cell–cell or cell–extracellular matrix (ECM) adhesion systems, the longevity of adhesion–receptor complexes is prolonged under tensile mechanical force through catch bonds. These bonds facilitate the capture or retention of cells under flow conditions while still permitting release under reduced mechanical force. For instance, vinculin, a protein, forms a force‐dependent catch bond with F‐actin via its tail domain, with bond lifetimes exhibiting a strong dependence on the direction of the applied force. This mechanism potentially contributes to the maintenance of front‐rear asymmetry in migrating cells. This part is described in detail in Section 5.2

#### 5.1.3 External Environment Regulates Biomolecular Mechanical Structure

Variations in the external chemical environment induce distinct changes in the mechanical properties of protein molecules.^[^ ^355^, ^394^ ^]^ Lei and her collaborators investigated the protein‐containing C1 structural domain in the gram‐positive bacterium *C. aerogenes* Cpe0147 and revealed that ester bonds lock the structure into a partially unfolded conformation, where the ester bonds are largely inaccessible to bulk water.^[^ ^403^ ^]^ Disrupting this structural correlation through cyclic arrangement results in sequential protein unfolding and ester bond hydrolysis. Conversely, disruption of the protein structure at alkaline pH or low calcium concentrations diminishes the protective effect and leads to ester bond hydrolysis. Further study demonstrated that ester bonds are chemically unstable yet mechanically stable, providing a basis for designing responsive materials using ester bonds as mechanically inert units.^[^ ^406^ ^]^

In living organisms, hydrophobic interactions play a huge role in the formation of self‐assembled structures for biomolecules such as proteins and polysaccharides, are challenging to study due to methodological difficulties at both macro‐ and microscopic scales. Di and his colleagues employed single‐molecule force extension curves to continuously monitor the size‐dependent hydrophobic free energy of polymer nanospheres. They observed a transition in the hydrophobic free energy of polymer nanospheres from a cubic to a square relationship within the 1 nm radius range, consistent with the Lum–Chandler–Weeks theory and simulation results. They discovered that the interaction strength was linearly related to the cation concentration, with the order of interaction strengths of different ions differing from that observed in the gas phase, possibly due to variations in the dehydration ability of the cations. These studies provide valuable insights and guidance for regulating chemical molecules to modulate mechanical properties.

#### 5.1.4 Materials Prepared Based on Biomechanochemistry

Furthermore, chemical modifications extensively influence majority of proteins post‐translational modification, thereby modulating their functions and fate within living organisms.^[^ ^439^, ^440^, ^441^ ^]^ These modifications are primarily catalyzed by specific enzymes that recognize target sequences in proteins. However, it is essential to note that the substrate must adopt a specific conformation to accommodate the enzyme binding site.^[^ ^442^, ^443^, ^444^ ^]^ Therefore, mechanical force can alter the conformation of the substrate, thereby regulating the activity of its enzyme.

Given the distinctive mechanical properties of proteins, there is a promising opportunity to utilize protein building blocks for the rational design of biomaterials, particularly hydrogels, with independently controllable key mechanical features such as Young's modulus, toughness, extensibility, and fatigue threshold. Since the inception of protein hydrogels aimed at mimicking the mechanical properties of muscles, numerous studies have explored protein‐based hydrogels.

**Figure 13:**
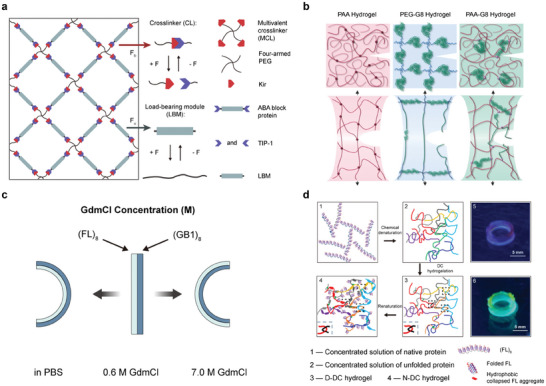
Building Biomaterials with Designed Proteins. a) Directed regulation of material properties through modulation of protein selection. Reproduced with permission.^[^ ^245^ ^]^ Copyright 2018, Springer Nature Limited. b) The material properties were altered by regulating the role played by proteins in the material. Reproduced with permission.^[^ ^244^ ^]^ Copyright 2020, Springer Nature Limited. c) Preparation of autonomous biomaterials with temporal regulation using differences in the mechanochemistry of different proteins in chemical environments. Reproduced with permission.^[^ ^445^ ^]^ Copyright 2022, Springer Nature Limited. d) Protein topological entanglements can be used to prepare extremely high‐strength biomaterials for bone repair. Reproduced with permission.^[^ ^446^ ^]^ Copyright 2023, Springer Nature Limited.

Proteins can absorb a significant energy during the unfolding process due to their rich internal mechanical structure, resulting in an effective delay in the diffusion of fracture energy. As depicted in **Figure** **13**a, Wu and his colleagues achieved notable advancements through rational design, wherein they effectively controlled the gel fracture position by modulating the unfolding force and the interplay between interprotein interactions and covalent interactions.^[^ ^245^ ^]^ This breakthrough has greatly enhanced our understanding of the relationship between the macroscopic mechanical properties of networks and microscopic mechanophores.

Achieving fatigue and fracture resistance through energy dissipation has become a common approach for enhancing the mechanical properties of materials. However, the persistent challenge of hysteresis complicates efforts to achieve optimal outcomes in bionic muscle or cardiac muscle repair. Lei and colleagues have addressed this issue by demonstrating anti‐fracture and relaxation uncoupling through the manipulation of proteins as cross‐linkers in the network. This modulation is attributed to differences in whether the resulting backbone polymers are entropy‐driven or enthalpy‐driven^[^ ^244^ ^]^ (Figure 13b). When used as a skeletal unit, proteins exhibit sufficient ductility during stretching, enabling significant energy dissipation with minimal deformation. Conversely, as crosslinks for long random chains, proteins demonstrate low hysteresis, directing all energy dissipation toward slowing crack propagation. This results in enhanced fatigue and fracture resistance.

Moreover, proteins exhibit similar persistence lengths but display contour lengths that can be influenced by the external environment. Li's team opted for two tandem modular elastin proteins, (GB1)_8_ and (FL)_8_, to construct a protein bilayer structure. Both protein layers exhibited distinct denaturation‐dependent swelling profiles and Young's moduli. Consequently, due to the swelling changes induced by protein defolding, the bilayer hydrogels demonstrated highly programmable and reversible bidirectional bending deformations contingent upon the denaturant concentration and layer geometry. Building upon these programmable bending behaviors, the team employed the protein bilayer structure as a hinge to facilitate 1‐D to 2‐D and 2‐D to 3‐D folding transitions in patterned hydrogels (Figure 13c).^[^ ^445^ ^]^ Recently, the team investigated how the seemingly contradictory properties of high stiffness, toughness, and fast recovery could be reconciled through the ability of proteins to unfold. They introduced chain entanglement into soft protein hydrogels using a ferredoxin‐like protein polyprotein, significantly enhancing their stiffness while preserving their toughness. This material offers a biomechanically compatible environment conducive to the regeneration of cartilage and osteochondral bone (Figure 13d).^[^ ^446^ ^]^

Similar to polymers, proteins can undergo structural changes induced by photostimulation. Wu and his colleagues devised a photocontrolled gel material utilizing Dronpa protein, enabling spatiotemporal modulation with minimal alterations to the external chemical environment.^[^ ^447^ ^]^ Additionally, Li and his team utilized LOVTRAP, composed of the protein LOV2 and its binding partner ZDark1, to develop a novel strategy for decorating/releasing fluorescent proteins in a light‐controlled and spatially defined manner on blank protein hydrogel slabs. This innovative approach provides a novel reversible information storage method for soft and wet materials, paving the way for the development of next‐generation protein‐based smart materials for information storage and anticounterfeiting applications.^[^ ^448^ ^]^

Proteins exhibit a diverse array of structures and interactions at multiple levels, encompassing polar, hydrophobic, and acid–base characteristics. Consequently, they demonstrate a wide range of mechanical responses under various external conditions, owing to the intricate interplay between their chemical environment and mechanical stimuli. The dynamic interplay between these factors and the resulting mechanistic responses presents a captivating area of inquiry.^[^ ^449^, ^450^ ^]^

### 5.2 Mechanical Regulation of Cellular Processes

The regulation of molecular mechanics is often investigated in vitro through single‐molecule force spectroscopy techniques, enabling precise control over experimental conditions. However, at the cellular level, mechanical forces must be transmitted between cells and their surrounding environment, which is typically composed of neighboring cells or the extracellular matrix (ECM).^[^ ^451^, ^452^, ^453^, ^454^, ^455^, ^456^, ^457^ ^]^ This intercellular force transmission is facilitated by specific proteins, notably integrins, which are instrumental in converting mechanical signals into biochemical cues. Consequently, the myriad ways in which cells sense mechanical forces and elicit various responses pose challenges for studying mechanical regulation at the cellular scale. The advent of biocompatible optical probes, including genetically encoded voltage indicators, molecular rotors, fluorescent dyes, semiconducting nanoparticles, and lanthanide‐doped upconverting nanoparticles, coupled with advancements in biomaterials, has enabled researchers to directly observe force transduction processes in vivo.^[^ ^32^ ^]^ Through these innovative approaches, scientists have made significant strides in elucidating how mechanical forces govern cellular functions and behaviors, contributing to a deeper understanding of mechanobiology.

#### 5.2.1 Biological Effects of Structural Transitions in Chemical Bonds at the Molecular Level

At the molecular level, structural changes in mechanochemistry can induce internal interactions within proteins, consequently leading to diverse cellular responses. Cysteine, the second most highly conserved amino acid after tryptophan in protein evolution,^[^ ^458^, ^459^ ^]^ plays a crucial role in these interactions. The breaking or reduction of disulfide bonds follows an SN2‐type nucleophilic substitution mechanism, where one bond is broken while another is formed synchronously, a process known as sulfur‐disulfide exchange, which exhibits high directionality.^[^ ^460^ ^]^ This exposure facilitates the formation of new hydrogen bonds, consequently lowering the pKa and enabling the chemical reaction to proceed.^[^ ^461^, ^462^, ^463^, ^464^, ^465^ ^]^

**Figure 14:**
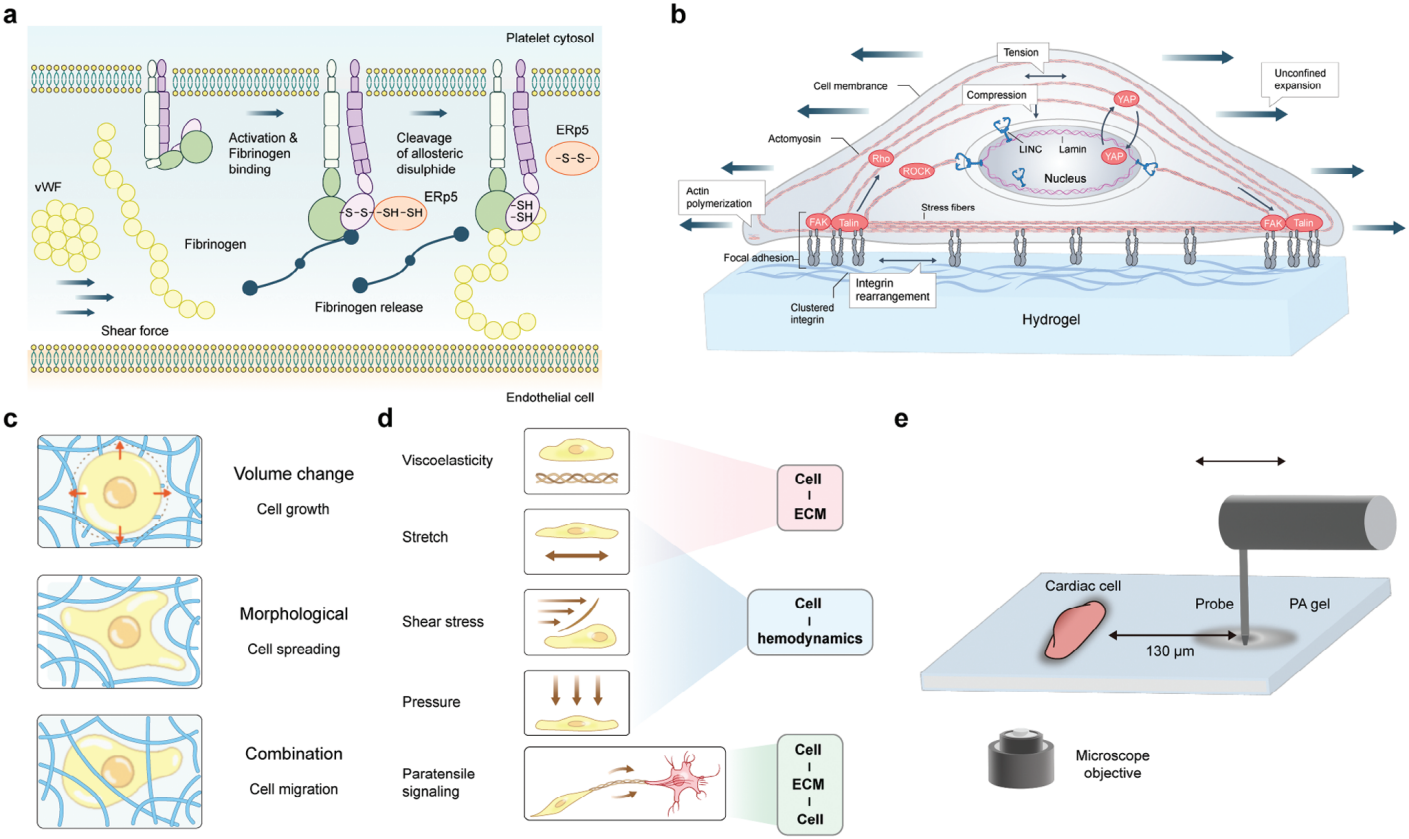
Mechanical regulation of the cellular environment. a) Structural transformation of isomeric disulfide bonds at high flow speeds prompts von Willebrand factor (vWF) to bind to integrins and accomplish platelet adhesion. Reproduced with permission.^[^ ^452^ ^]^ Copyright 2019, Springer Nature Limited. b) The mode of mechanotransduction of cells with the ECM in a 2‐D matrix is highly correlated with several force transducer proteins. Reproduced with permission.^[^ ^476^ ^]^ Copyright 2020, Springer Nature Limited. c) 3‐D cellular and ECM mechanotransduction modes with corresponding cellular behavior, and cellular behavior is highly correlated with the viscoelasticity and viscoplasticity of the material. Reproduced with permission.^[^ ^453^ ^]^ Copyright 2020, Springer Nature Limited. d) Cells interact with the ECM through interactions that lead to crosstalk, resulting in outcomes. Reproduced with permission.^[^ ^455^ ^]^ Copyright 2022, Elsevier. e) Rhythmic stimulation of cardiomyocytes can cause the cells to vibrate at the same frequency.

Mechanical forces can induce distortions in the geometry of substrate disulfide bonds, including twisting, bending, or stretching of the sulfur–sulfur bond. These distortions facilitate the alignment of sulfur atoms and promote sulfur‐disulfide exchange. In essence, the mechanochemical coupling of thiol‐disulfide exchange allows the disulfide bond to function as a molecular switch for mechanical signaling within the proteins housing it. Disulfide chemistry plays a crucial role in regulating the function of coagulation factors, such as tissue factor, factor XI, and vitronectin, thus mediating clot formation and hemostasis.^[^ ^466^, ^467^ ^]^ However, until recently, the interaction between disulfide bonds and mechanical forces in regulating platelet adhesion has just been identified^[^ ^452^ ^]^ (**Figure** **14**a). Oxidoreductases such as ERp5, are required for thrombosis after vascular injury, although the mechanisms controlling them remain unclear.^[^ ^468^, ^469^, ^470^, ^471^, ^472^, ^473^ ^]^ Among these, integrin αIIbβ3 is the most abundant receptor on the platelet surface. In its resting state, inactive αIIbβ3 adopts a bent conformation, concealing the head of the ligand interaction. Upon agonist activation, αIIbβ3 transitions from a bent to a stretched conformation, exposing its head. This disulfide bond is cleaved by secreted platelet ERp5.^[^ ^474^, ^475^ ^]^ This observation suggested that stretching of the disulfide bond, induced by either ligand binding or mechanical force, is necessary for ERp5 activity.

#### 5.2.2 Biological Effects Due to Synergistic Interaction of Mechanophores at the Single‐Cell Level

In single‐cell force signaling perception, protein mechanical structures and interactions play a pivotal role^[^ ^476^ ^]^ (Figure 14b). Early literature extensively documents the intricate relationship between various cellular functionalities—such as differentiation, spreading, and migration—and the mechanical stimuli originating from the external environment. Cellular responsiveness to external mechanical cues often stems from localized protein molecules influenced by forces, engaging in mechanotransduction processes that convert mechanical signals into biochemical cues. This cascade of events ultimately results in dynamic alterations in cell behavior over both short and extended time frames. Proteins responding to mechanical signals typically undergo structural domain unfolding, thereby exposing hidden recognition sites or losing existing ones.^[^ ^477^, ^478^, ^479^ ^]^ For instance, focal adhesion kinase (FAK) exposes phosphorylation sites in response to external forces, leading to their phosphorylation and the formation of molecular clutch structures that induce cell‐substrate interactions and feedback mechanisms.^[^ ^480^, ^481^, ^482^, ^483^, ^484^, ^485^, ^486^ ^]^ Similarly, the talin rod structural domain R3 requires folding of the structural domain to interact with RIAM.^[^ ^487^, ^488^ ^]^ Studies have demonstrated that the unfolding and folding of different structural domains of talin influence the function of other domains, accomplish diverse cellular signaling pathways, and alter the force chemistry of the protein.^[^ ^203^, ^417^, ^418^, ^437^, ^489^ ^]^

Catch bonds, which exhibit longer lifetimes at higher external forces, play a crucial role in mediating interactions between cellular functions and mechanical stimuli, exerting multiple effects on cell behavior and adhesion.^[^ ^490^ ^]^ Through these interactions, integrins facilitate hardness measurements of the cell substrate, prompt cells to migrate toward the hard substrate, and induce YAP/TAZ migration toward the nucleus, leading to nuclear translocation and the cellular perception of external mechanical stimuli.^[^ ^203^, ^486^, ^487^, ^491^, ^492^, ^493^, ^494^, ^495^, ^496^ ^]^ Similarly, the Rho‐kinase pathway yields different outcomes in response to positive and negative stimuli, suggesting the presence of Catch bonds in the FAK cellular force transduction pathway as well.^[^ ^497^ ^]^ Overall, rapid conformational changes, which are sensitive to force, enable these proteins to function as responsive mechanical switches. Forces acting upon proteins profoundly influence their interactions, potentially facilitating reactions that were previously considered improbable. Notably, the polymerization and extension of actin, which are crucial for cellular processes such as spreading, adhesion, and migration, are intricately dependent on the forces applied to actin filaments. Even a modest force of 6 pN can induce a nearly eightfold difference in extension velocity, highlighting the sensitivity of these processes to mechanical cues.^[^ ^498^ ^]^

#### 5.2.3 Biological Effects of Culture Materials Due to Viscoelasticity and Viscoplasticity at the Macro Level

Another crucial aspect of bio‐mechanical chemistry in studying mechanoregulation is the exploration of cell‐ECM interactions under multicellular conditions, which are closely linked to viscoelasticity and viscoplasticity induced by mechanochemistry or physical entanglement.^[^ ^476^, ^499^, ^500^, ^501^ ^]^ During various biological processes, as cells exert forces on the ECM through pushing and pulling, the ECM initially resists these actions, a resistance primarily governed by the stiffness of the ECM.^[^ ^502^, ^503^, ^504^ ^]^ As cell growth leads to volume changes in the ECM, the strength of these pushing and pulling forces typically increases, resulting in nonlinear increases in ECM resistance or stiffening, a phenomenon termed nonlinear elasticity. However, with continued pushing and pulling over time, the resistance of the ECM to cell‐induced deformation gradually relaxes due to stress relaxation, and the ECM exhibits creep under loading, illustrating viscoelastic behavior. Eventually, due to the mechanical plasticity of the ECM, permanent deformation occurs when the cell releases force, facilitating cell spreading as the ECM expands. These two properties of the ECM—viscoelasticity and viscoplasticity—are combined when cells migrate. Moreover, cell viability is influenced by the ECM pore size, which affects the cell migration pattern^[^ ^453^ ^]^ (Figure 14c). The interplay between these mechanical properties and cellular behavior is critical. These diverse mechanical properties of the cell significantly impact intracellular signaling, transcription, and phenotype through feedback from the ECM. Consequently, cells sense and respond to mechanical signals provided by the ECM, a process known as cell‐ECM mechanotransduction.

Cell–ECM interactions play important roles in various cellular functions, such as survival, proliferation, migration, and differentiation, and these interactions can be mechanically transmitted to fulfill numerous functions. For instance, cell‐extracellular matrix (ECM) crosstalk can result in cross‐linking and deposition of ECM, leading to increased elasticity and decreased viscosity. Cell‐hemodynamic crosstalk exposes cells to shear and pressure, activating mechanical pathways. On the other hand, cell‐ECM‐cell crosstalk enables paraneural signaling to activate myofibroblasts (parallel tensile signaling)^[^ ^505^ ^]^ and induce ECM accumulation^[^ ^455^ ^]^ (Figure 14d). Therefore, it is necessary to synthesize biomaterials, particularly hydrogels, to mimic the ECM in vitro. Cells are cultured in hydrogels, and the mechanical properties of the hydrogels or the application of mechanical external forces are utilized to modulate the function and behavior of the cells. Excellent progress has been made in the last decade.^[^ ^447^, ^506^, ^507^, ^508^, ^509^, ^510^ ^]^ Hydrogels with faster stress relaxation resulted in greater bone regeneration.^[^ ^511^ ^]^ The advantageous effects of hydrogels in various applications, including cartilage regeneration, vocal cord regeneration, and improved pathological remodeling after myocardial infarction, are attributed to their viscoelastic properties.^[^ ^512^, ^513^, ^514^, ^515^ ^]^ Additionally, in vitro intestinal organoid formation and other applications for promoting skeletal muscle, liver, and neurological organoid formation have been achieved through viscoelastic hydrogels.^[^ ^516^, ^517^, ^518^, ^519^ ^]^ Several early studies concluded that more rapidly degrading hydrogels led to greater tissue regeneration than more slowly degrading gels.^[^ ^520^, ^521^, ^522^ ^]^

Most research in this area has focused on the interactions between cells and the extracellular matrix (ECM). However, a recent study reported a surprising phenomenon: mechanical communication in cardiac cells leads to synchronized beating. In this study, it was observed that an isolated cardiac cell can be trained to beat at a given frequency by mechanically stimulating the underlying substrate.^[^ ^523^ ^]^(Figure 14e) Deformations are induced using an oscillatory mechanical probe that mimics the deformations generated by a beating neighboring cardiac cell.^[^ ^524^, ^525^, ^526^ ^]^ Unlike electrical field stimulation, the probe‐induced beating rate is maintained by the cell for an hour after the stimulation stops, suggesting that long‐term modifications occur within cells. This finding represents the first demonstration of direct mechanical communication between cells.

Presently, the expanding realm of biomechanochemistry research, transitioning from 2‐D to 3‐D hydrogel cell culture and from phenomena elicited by mechanical physical cues to indispensable mechanical force transduction pathways, has facilitated a profound and enhanced comprehension of the role of mechanical signals in both developmental processes and pathological conditions.^[^ ^38^, ^527^, ^528^, ^529^, ^530^, ^531^ ^]^ Undoubtedly, the quantification of forces and fields within biological systems holds paramount importance in elucidating physiological phenomena and understanding the mechanisms underlying various pathological conditions. Nonetheless, a persistent challenge persists in the inconsistency and conflicts observed in the results obtained through diverse measurement techniques. Despite these challenges, the study of biomechanochemistry remains of paramount significance within the biomedical domain. Its implications span critical areas such as cancer metastasis, immune function, maintenance of stemness, and the differentiation of stem cells, all of which are central to advancements in tissue engineering and regenerative medicine.^[^ ^532^, ^533^ ^]^ Thus, addressing the existing discrepancies and refining measurement methodologies are imperative for furthering our understanding and leveraging the potential of biomechanochemistry in biomedical research and applications.^[^ ^534^, ^535^, ^536^, ^537^, ^538^, ^539^, ^540^ ^]^

## 6 Conclusion and Outlook

After years of development, mechanochemistry has become an important research direction that spans across multiple disciplines, including physics, chemistry, biology, and materials science. Substantial process has been achieved in both mechanism studies and applications.^[^ ^541^, ^542^, ^543^, ^544^, ^545^, ^546^, ^547^ ^]^ However, there are many challenges remained in mechanochemistry. One of the significant challenges in mechanochemistry is elucidating the precise mechanisms by which mechanical force induces chemical transformations. Unlike reactions driven by heat or light, where energy absorption and transfer can be relatively easily monitored, the transient and localized nature of mechanochemical activation makes it difficult to observe directly. Therefore, how to achieve more efficient detection methods with superhigh temporal and spatial resolution and controllability are key issues in the current experimental setup.

On the other hand, as scientific research deepens, people's understanding of physical and chemical processes has become more detailed and accurate, and more and more novel phenomena and substances have been discovered. For example, “opposite charges attract, like charges repel” is a basic principle of fundamental physics. However, a new study by Madhavi Krishnan et al. from University of Oxford has overturned this basic principle of physics, proving that particles with similar charges can attract each other in a solution, and the effects between positive and negative charges vary with the solvent.^[^ ^548^ ^]^ These novel phenomena may also pose new challenges for the study of the mechanochemical reaction mechanism. There is a spiral relationship between devices, theories and experiments, and progress in all three is indispensable.

Thus, one of the future directions in mechanochemistry is the development of novel instrumentation for precise control and measurement of mechanical forces, as well as the chemical analysis. Although current single‐molecule technologies are already very powerful, capable of exploring the molecular mechanisms of mechanochemical reactions at the molecular level, more technologies are still needed to study their mechanisms.^[^ ^66^, ^549^, ^550^, ^551^ ^]^ Among them, Raman spectroscopy, as a molecular vibration detection technology, is one of the potential technologies. Recently, Tahei Tahara et al. observed the ultrafast transient of trans and cis forms within a single molecule using ultrafast Raman spectroscopy with time resolution of 0.1 ps.^[^ ^552^ ^]^ Combining with the technique of applying mechanical force such as AFM, the ultrafast Raman‐AFM would be helpful for mechanochemistry study.^[^ ^553^ ^]^ AFM‐based infrared spectroscopy (IR) is another rapidly emerging technique that provides chemical analysis and compositional mapping with spatial resolution far below conventional optical diffraction limits.^[^ ^554^, ^555^, ^556^, ^557^, ^558^, ^559^ ^]^ These techniques can enrich our understanding the mechanism of mechanochemistry in molecular level.

Many physical and chemical processes last only on the attosecond scale, meaning these phenomena occur instantaneously, which is difficult to clearly observe these processes. However, the advent of attosecond laser technology which was awarded by Nobel Prize in physics 2023 has given us the means to observe and study these fleeting events as quickly as they happen.^[^ ^560^, ^561^, ^562^ ^]^ Within atoms and molecules, the movement and state transitions of electrons can occur on an attosecond time scale. For example, electron transitions, ionization of electrons, and their recombination processes, etc. Similar examples include certain initial stages of chemical reactions, such as electron transfer; the interaction between light and matter, such as light‐induced electron excitation, ionization, and other nonlinear optical effects; in quantum systems, coherence and decoherence processes may occur on an attosecond time scale, affecting the evolution of the quantum state of the system.^[^ ^563^, ^564^, ^565^, ^566^ ^]^ Furthermore, attosecond lasers can also be used to study fast chemical reactions, such as observing and controlling the process of bond breaking and bond formation in chemical reactions, thereby providing new possibilities for understanding and controlling chemical reactions, as well as important information for the development of new materials and technologies.^[^ ^567^, ^568^, ^569^, ^570^, ^571^, ^572^ ^]^ Attosecond laser technology has opened a new window for us, enabling us to study and control the microscopic processes of matter on an unprecedented time scale, thereby revealing and understanding the fundamental properties and behavioral patterns of matter.

Advances in nanotechnology and high‐speed imaging techniques are expected to enable higher resolution and faster real‐time monitoring technologies.^[^ ^573^, ^574^, ^575^, ^576^ ^]^ This would allow us to observe and understand changes at the molecular and atomic levels under the influence of mechanical forces with greater precision.^[^ ^576^, ^577^, ^578^, ^579^ ^]^ The next step is the integration of multi‐scale simulations and experiments. Combining molecular dynamics simulations and quantum chemical calculations with experimental data will become more refined. With the development of computational chemistry tools, predicting and designing mechanochemical reaction pathways will become more feasible.^[^ ^580^, ^581^, ^582^ ^]^ This will help in understanding complex mechanochemically induced processes and guide the development of new materials and reactions.

In addition to the traditional calculation methods in mechanochemistry, the Artificial Intelligence (AI) techniques represents a burgeoning area of research, poised to revolutionize how we understand and manipulate chemical reactions under mechanical forces. AI algorithms, particularly machine learning (ML) and deep learning (DL), can predict the outcomes of mechanochemical reactions with high accuracy. By training on datasets of known mechanochemical reactions, AI models can learn the underlying patterns and predict the behavior of new, untested reactions. This capability is invaluable for identifying promising reaction pathways or materials with specific properties without the need for extensive physical experimentation. AI can optimize mechanochemical reaction conditions, such as pressure, temperature, and grinding duration, to maximize yield or achieve desired material characteristics. AI techniques aid in the design and discovery of new materials through mechanochemistry.^[^ ^583^, ^584^ ^]^ This approach accelerates the pace of discovery and can lead to the development of materials with enhanced performance or entirely new functionalities.^[^ ^585^ ^]^ Also, AI can help us to design different kinds of mechanophores.

Based on these advanced analytical techniques, including in situ monitoring of reactions and computational modeling, the mechanochemistry would become a much more vibrant field and promise significant applications. For example, the piezolelctric catalysis in solid‐state organic mechanochemistry;^[^ ^586^, ^587^, ^588^, ^589^, ^590^, ^591^, ^592^ ^]^ nanoimprint in polymer mechanochemistry, demonstrated by Canon's nanoimprint lithography (NIL), is poised to revolutionize the semiconductor industry;^[^ ^593^, ^594^, ^595^, ^596^ ^]^ the brain‐computer interface is important in interface mechanochemistry;^[^ ^597^, ^598^ ^]^ the force squeeze and pull life in biomechano‐chemistry.^[^ ^599^, ^600^, ^601^, ^602^ ^]^ As research in these areas continues to evolve, we can anticipate novel materials, greener chemical processes, and innovative biomedical applications that leverage the unique properties of mechanochemical reactions.^[^ ^603^, ^604^, ^605^ ^]^ The future of mechanochemistry promises not only to expand our understanding of chemical and physical phenomena but also to catalyze a transition towards more sustainable and efficient practices across scientific disciplines and industries.

Advances in single‐molecule measurement devices, including metamaterials capable of electrically induced and optically induced magnetic responses, hold immense promise for precise manipulation and characterization at the nanoscale.^[^ ^606^ ^]^ Notably, single‐molecule measurements have broader implications, extending into scientific and technological realms. For instance, optical tweezers, when used alongside arrays of neutral atoms, enable robust quantum information storage and manipulation, including the generation of entangled states crucial for quantum computing applications.^[^ ^607^ ^]^ Furthermore, mechanochemistry finds compelling applications in medical treatments, such as the use of tapered optical fiber tweezers (TOFTs).^[^ ^608^ ^]^ These versatile tools offer noninvasive manipulation of cells within diverse biological microenvironments, facilitating single‐cell manipulation and neuronal repair.

Mechanochemistry, while not novel in origin, has emerged as a cornerstone of contemporary scientific exploration due to its profound implications and the intricate diversity of underlying mechanisms involved. Comprehending the molecular intricacies of mechanochemical processes necessitates a synthesis of principles from mechanics and physics, facilitating the formulation of robust theoretical frameworks and sophisticated simulation methodologies. In broadening the scope of mechanochemistry, collaboration across disciplines is imperative, drawing upon insights from engineering, material science, and biomedicine. This interdisciplinary synergy not only propels the translation of fundamental discoveries into practical applications but also opens new avenues for exploration and innovation. The trajectory of scientific inquiry in mechanochemistry is focused on the development of environmentally sustainable, efficient, and precise methodologies. By harnessing the inherent potential of mechanochemistry, researchers endeavor to address pressing societal challenges and catalyze transformative advancements in scientific research and technological innovation.

## Conflict of Interest

The authors declare no conflict of interest.
